# Combined Fat Mass and Fat-free Mass Indices and Lung Function Among Japanese Population: The Tohoku Medical Megabank Community-based Cohort Study

**DOI:** 10.2188/jea.JE20220355

**Published:** 2024-03-05

**Authors:** Masato Takase, Mitsuhiro Yamada, Tomohiro Nakamura, Naoki Nakaya, Mana Kogure, Rieko Hatanaka, Kumi Nakaya, Ippei Chiba, Ikumi Kanno, Kotaro Nochioka, Naho Tsuchiya, Takumi Hirata, Yohei Hamanaka, Junichi Sugawara, Tomoko Kobayashi, Nobuo Fuse, Akira Uruno, Eiichi N. Kodama, Shinichi Kuriyama, Ichiro Tsuji, Atsushi Hozawa

**Affiliations:** 1 Graduate School of Medicine, Tohoku University, Sendai, Japan; 2 Tohoku Medical Megabank Organization, Tohoku University, Sendai, Japan; 3 Tohoku University Hospital, Tohoku University, Sendai, Japan; 4 Institute for Clinical and Translational Science, Nara Medical University, Nara, Japan; 5 International Research Institute of Disaster Science, Tohoku University, Sendai, Japan

**Keywords:** adipose tissue, body composition, epidemiology, lung function, obesity

## Abstract

**Background:**

Although fat mass index (FMI) and fat-free mass index (FFMI) affect lung function, FMI and FFMI are not independent of each other, since FMI and FFMI were calculated as fat mass and fat-free mass divided by height squared, respectively. We aimed to examine the association of combined FMI and FFMI with lung function.

**Methods:**

In this cross-sectional study, lung function was evaluated using forced expiratory volume at 1 s (FEV_1_) and forced vital capacity (FVC) measured using spirometry. Both FMI and FFMI were classified into sex-specific quartiles (16 groups). Analysis of covariance was used to assess the associations of combined FMI and FFMI with lung function. The trend test was conducted by stratifying the FMI and FFMI, scoring the categories from 1–4 (lowest–highest), and entering the number as a continuous term in the regression model.

**Results:**

This study included 3,736 men and 8,821 women aged ≥20 years living in Miyagi Prefecture, Japan. The mean FEV_1_ was 3.0 (standard deviation [SD], 0.7) L for men and 2.3 (SD, 0.5) L for women. The mean FVC was 3.8 (SD, 0.7) L for men and 2.8 (SD, 0.5) L for women. FMI was inversely associated with lung function among all FFMI subgroups in both sexes. Conversely, FFMI was positively associated with lung function in all FMI subgroups in both sexes.

**Conclusion:**

Higher FMI was associated with lower lung function independent of FFMI; higher FFMI was associated with higher lung function independent of FMI. Reducing FMI and maintaining FFMI might be important for respiratory health.

## INTRODUCTION

Lung function, indicated by forced expiratory volume in 1 s (FEV_1_) and forced vital capacity (FVC) measured using spirometry, are significant predictors of future cardiovascular disease and mortality among the general population.^1^^–^^3^ Therefore, maintaining lung function may be important to prevent chronic lung disease, as well as cardiovascular disease and premature death.

Body mass index (BMI) is widely used to assess obesity and is inversely associated with lung function and volume.^4^^–^^6^ However, BMI may be an imperfect indicator of adiposity because it cannot distinguish between fat mass (FM) and fat-free mass (FFM).^7^^,^^8^ FM may have negative proinflammatory effects on lung function and impede expansion and excursion of the diaphragm and chest wall.^5^^,^^6^ Conversely, FFM may reflect increased diaphragm and chest wall strength during expansion and contraction during breathing, which may contribute to increased lung function.^9^ Therefore, since FM and FFM play different roles in lung function, understanding their association with lung function may be achieved by considering body composition rather than BMI.

Many previous studies have examined the association between body composition and lung function and found that FM was linearly and inversely associated with lung function,^10^^–^^21^ whereas FFM was linearly and positively associated with lung function.^10^^–^^13^^,^^17^^,^^19^^–^^21^ These findings suggest that FM and FFM may be linearly associated with lung function. Although many previous studies have used absolute FM, FFM, and percentage of body fat (BF%) as indicators of body composition,^10^^,^^12^^,^^14^^–^^18^^,^^20^^,^^21^ absolute FM and FFM are correlated with height. The BF% is affected by FFM because the weight is equal to the sum of FM and FFM.^7^ Moreover, since there is a correlation between FM and FFM because a higher FFM is required to carry excess increased body fat, the estimation of results may be unstable if we adapt FM and FFM to the same statistical model simultaneously. Therefore, clarifying the effects of FM and FFM on lung function is difficult.

The fat mass index (FMI) and fat-free mass index (FFMI), calculated as the FM and FFM in kilograms divided by the height in meters squared, respectively, have been proposed as indicators of body composition.^7^^,^^8^ These indices are unaffected by each other and are useful for comparing individuals of different heights.^7^^,^^8^ However, since height is included as a denominator in the formulas for these indices, FMI and FFMI are not independent of each other. Therefore, it is difficult to adapt both indicators simultaneously in the same statistical model. When we combined FMI and FFMI, we could identify individuals with higher FM and lower FFM and individuals with lower FM and higher FFM. Furthermore, this strategy not only avoids multicollinearity but also reveals the association between FMI and health outcomes in each FFMI subgroup and the association between FFMI and health outcomes in each FMI subgroup.^22^^–^^24^

To the best of our knowledge, no study has examined the association of combined FMI and FFMI with lung function. Therefore, to clarify the association between body composition and lung function, we assessed the cross-sectional association of combined FMI and FFMI with lung function in a Japanese population using data from the Tohoku Medical Megabank Community-Based Cohort Study.

## METHODS

### Study design and population

We conducted a cross-sectional study using data from the Tohoku Medical Megabank Community-Based Cohort Study, the design of which has been described in detail elsewhere.^25^^,^^26^ We used three approaches to recruit participants. The type 1 survey (40,433 participants) was conducted at specific municipal health check-up sites. The type 1 additional survey (664 participants) was conducted on different dates from specific municipal health checkup data. The type 2 survey (13,855 participants) was conducted at the Community Support Center. In all three approaches, the same basic information from blood and urine test results, a questionnaire, and municipal health check-ups was collected.^26^ Physiological measurements were only obtained in the type 2 survey (eg, axial length, body composition, spirometry, bone density measurement, carotid ultrasound imaging, home blood pressure, hearing acuity, oral examination). The source population for the study comprised men and women aged ≥20 years living in Miyagi Prefecture, northeastern Japan. Informed consent was obtained from 54,952 participants. All participants were recruited from May 2013 to March 2016. This study was conducted in accordance with the amended Declaration of Helsinki. Local institutional review boards or independent ethics committees approved the protocol, and written informed consent was obtained from all patients. This study was approved by the Institutional Review Board of the Tohoku Medical Megabank Organization (approval number: 2021-4-028, approval data: May 31, 2021).

Participants were required to undergo several physiological measurements, including body composition and lung function, for inclusion in the analysis. Therefore, we only used data from participants in the type 2 survey (13,855 participants). We excluded participants who withdrew from the study by July 13, 2021; failed to return the self-reported questionnaire; did not undergo physiological measurements; or had missing data regarding height, weight, BF%, FEV_1_, FVC, and vital capacity (*n* = 1,298). Ultimately, data from 12,557 participants (3,736 men and 8,821 women) were analyzed.

### Anthropometry

Height was measured using a stadiometer (AD-6400; A&D Co. Ltd., Tokyo, Japan). Weight and BF% were measured using a body composition analyzer (InBody720; Biospace Co, Ltd, Seoul, Korea). To account for the weight of the participant’s clothing, 1.0 kg was deducted from the measured weight. BMI was calculated as weight (kg) divided by height squared (m^2^). FM was calculated by multiplying the weight by the BF%. Then FMI was calculated as FM (kg) divided by height squared (m^2^). Similarly, the FFM% was calculated by subtracting the BF% from 100%; the FFM was then calculated by multiplying the weight by the FFM%. Subsequently, FFMI was calculated as FFM (kg) divided by height squared (m^2^).^7^^,^^8^^,^^22^^–^^24^

### Lung function assessment

Lung function parameters, including FEV_1_, FVC, and vital capacity, were measured using a spirometer (HI-801; Chest M.I., Inc., Tokyo, Japan). Spirometry was performed with participants in a seated position using an attachable nose clip. All test results were regularly checked by trained physicians in a blind manner, and invalid data were excluded from the data set. We used FEV_1_ and FVC as outcomes because FEV_1_ and FVC are predictors for mortality and cardiovascular disease in the general population.^1^^–^^3^ The percentage of vital capacity was predicted according to age, sex, and height. Restrictive ventilatory impairment was defined as a reduced vital capacity of <80% of the predicted value, and obstructive ventilatory impairment was defined as a reduced FEV_1_/FVC of <70%.

### Other measurements

The participants’ demographic characteristics, smoking status, and history of respiratory disease were obtained using a self-reported questionnaire. Smoking status was classified into five categories: never smokers (smoked <100 cigarettes in their lifetime), ex-smokers (smoked ≥100 cigarettes in their lifetime and were not current smokers), current smokers 1–19/day (smoked ≥100 cigarettes in their lifetime and currently smoke 1–19 cigarettes per day), current smokers ≥20/day (smoked ≥100 cigarettes in their lifetime and currently smoke ≥20 cigarettes per day), and unknown status.^27^ Passive smoking was defined as participants who answered that they had been exposed to the smoke from others at work or home on most days during the previous year. Drinking status was classified into five categories: never drinker (consumed little or no alcohol or was constitutionally incapable of alcohol consumption), ex-drinker (stopped drinking alcohol), current drinker (<23 or ≥23 g/day), and unknown. To calculate the amount of ethanol, the alcohol type was classified into six categories: sake, distilled spirits, shochu-based beverages, beer, whiskey, and wine. Alcohol consumption frequency was also classified into six categories: almost never, 1–3 days/month, 1–2 days/week, 3–4 days/week, 5–6 days/week, or daily. The amount of ethanol was determined by multiplying the type of alcohol by its frequency and amount. We set the cutoff value at 23 g as it is the most common unit for measuring the amount of alcohol consumed in Japanese people.^28^ Education level was classified as: below high school; vocational school, junior college, or technical college; university or graduate school; other; and unknown. Participants also indicated if they had a history of respiratory disease (asthma, chronic bronchitis, or chronic obstructive pulmonary disease [COPD]).

### Statistical analysis

Data are presented as means (standard deviations [SDs]) or medians (interquartile ranges) for continuous variables and as numbers (%) for categorical variables. All analyses were performed separately for men and women because the distributions of FMI, FFMI, and lung function vary by sex. The FMI was categorized into the following sex-specific cutoff points for quartiles of men: Q1, <4.3; Q2, 4.3–5.4; Q3, 5.5–7.1; and Q4; ≥7.1 kg/m^2^. For women the cutoff points were: Q1, <5.1; Q2, 5.1–6.6; Q3, 6.7–8.6; and Q4, ≥8.6 kg/m^2^. The FFMI was also categorized into the following sex-specific cutoff points for quartiles of men: Q1, <17.0; Q2, 17.0–17.9; Q3, 18.0–18.9; and Q4; ≥18.9 kg/m^2^. For women the cutoff points were: Q1, <14.5; Q2, 14.5–15.1; Q3, 15.2–16.0; and Q4, ≥16.0 kg/m^2^. To confirm the correlation coefficients between FMI and FFMI, we calculated the Pearson correlation coefficients.

Regarding the characteristics of the FMI quartiles, a trend test was performed on continuous variables using a simple linear model to evaluate linearity. We also performed a chi-square test to compare the characteristics of the categorical variables among the FMI quartiles. Furthermore, we performed a similar analysis to evaluate the linear associations and compare the characteristics of the categorical variables among the quartile groups for FFMI.

Analysis of covariance was used to calculate the least squares means of lung function indices (FEV_1_ and FVC) according to the FMI category. In the multivariable model, we adjusted for age, education level (below high school; vocational school, junior college, or technical college; university or graduate school; other; unknown), smoking status (never smoker, ex-smoker, current smoker [1–19 or ≥20 cigarrettes/day], unknown), passive smoking (yes, no), and drinking status (never drinker, ex-drinker, current drinker [<23 or ≥23 g/day], unknown). The association between FFMI and lung function indices was examined using analysis of covariance and the same models. The *P*-values for the analysis of linear trends were calculated by scoring the categories from 1 (the lowest category) to 4 (the highest category) and entering the number as a continuous term in the regression model. To confirm the robustness of the results, we performed multiple regression analysis using FMI as a continuous variable. Also, we conducted multiple regression analysis using FFMI as a continuous variable to investigate the linear association between FFMI and lung function.

We combined the quartile groups for FMI and FFMI and classified them into 16 groups. We also used an analysis of covariance to calculate the least squares means of the lung function indices for the 16 groups. To show the linear association between lung function with FMI among FFMI subgroups, we conducted multiple regression analysis by stratifying the FFMI and using FMI as a continuous variable in the regression model. Similarly, we also performed multiple regression analysis by stratifying the FMI and using FFMI as a continuous variable in the regression model.

Several sensitivity analyses were performed to test the robustness of our findings. First, to rule out the effect of smoking, the analysis excluded participants who were current smokers, ex-smokers, and unknown. Second, we performed stratified analyses according to age tertiles (men: <58, 58–67, and ≥67 years and women: <52, 52–64, and ≥64 years). Third, to eliminate the effect of respiratory disease, we selected only participants without restrictive ventilatory impairment, obstructive ventilatory impairment, and history of asthma, chronic bronchitis, or COPD.

A two-sided *P* < 0.05 was considered statistically significant. All analyses were performed using R software version 4.1.2 (R Foundation for Statistical Computing, Vienna, Austria).

## RESULTS

A total of 3,736 men and 8,821 women fulfilled all inclusion criteria and their data were included in the analyses. The mean age of the study participants was 60.1 (SD, 14.0) years for men and 56.2 (SD, 13.4) years for women. The FMI was higher in women than that in men, whereas the FFMI was higher in men than that in women. The mean FEV_1_ was higher in men (3.0; SD, 0.7 L) than that in women (2.3; SD, 0.5 L). Similarly, the mean FVC was higher in men (3.8; SD, 0.7 L) than that in women (2.8; SD, 0.5 L). Participants with higher FMI had higher BMI and FFMI and shorter height, FEV_1_, and FVC (eTable 1). Men with a higher FFMI were younger and shorter. Among both men and women, participants with higher FFMI had a higher BMI, FMI, FEV_1_, and FVC (eTable 2). The correlation coefficient between the FMI and FFMI was *r* = 0.37 (*P* < 0.001) for men and *r* = 0.51 (*P* < 0.001) for women.

The participant characteristics according to FMI and FFMI combined are shown in Table 1 and Table 2 for men and women, respectively. Men and women with higher FMI and FFMI were more likely to have a higher BMI. Men categorized as FMI Q1 and FFMI Q4 (the lowest FMI quartile and highest FFMI quartile, respectively) were younger, taller, and had higher FEV_1_, FVC, and percentage of vital capacity, and were more likely to be current smokers. Men categorized as FMI Q4 and FFMI Q1 (the highest FMI quartile and lowest FFMI quartile, respectively) were older, shorter, and had lower FEV_1_, FVC, percentage of vital capacity, and were less likely to be smokers. Similarly, women categorized as FMI Q1 and FFMI Q4 were taller and had higher FEV_1_, FVC, and percentage of vital capacity, and more likely to be current smokers. Women categorized as FMI Q4 and FFMI Q1 were older, shorter, and had lower FEV_1_ and FVC, and were less likely to be current smokers.

**Table 1.** Characteristics of combined FM and FFMI among men

FMI	Q1				Q2				Q3				Q4				All participants

FFMI	Q1	Q2	Q3	Q4	Q1	Q2	Q3	Q4	Q1	Q2	Q3	Q4	Q1	Q2	Q3	Q4	
Number	370	246	188	130	235	293	243	163	203	217	272	242	119	184	233	398	3,736
Age, years	59.9 (15.5)	57.4 (15.2)	55.2 (14.3)	49.2 (15.1)	64.5 (13.5)	61.4 (12.7)	58.6 (13.1)	54.7 (13.2)	66.5 (11.2)	63.7 (12.3)	61.1 (12.4)	56.3 (12.5)	71.2 (10.5)	66.6 (11.2)	63.2 (12.2)	55.6 (13.6)	60.1 (14.0)
Height, cm	167.8 (6.7)	168.1 (6.2)	169.5 (6.7)	170.9 (6.6)	166.0 (6.1)	167.8 (6.3)	167.7 (6.0)	169.5 (5.8)	165.4 (5.8)	165.9 (5.9)	167.5 (5.9)	169.3 (5.9)	162.6 (5.8)	164.6 (5.9)	166.1 (5.7)	169.3 (6.0)	167.5 (6.3)
BMI, kg/m^2^	19.2 (1.2)	20.8 (0.8)	21.8 (0.7)	23.0 (1.7)	21.1 (0.8)	22.4 (0.5)	23.3 (0.4)	24.7 (0.8)	22.5 (0.8)	23.7 (0.5)	24.7 (0.5)	26.1 (0.9)	24.6 (1.2)	25.7 (1.2)	26.7 (1.2)	29.3 (2.6)	23.8 (3.1)
BF%, %	16.3 (3.4)	16.1 (2.9)	15.7 (2.5)	14.6 (3.0)	23.3 (1.4)	22.0 (1.4)	21.1 (1.3)	20.1 (1.3)	27.7 (1.6)	26.3 (1.4)	25.3 (1.3)	24.1 (1.4)	33.9 (2.9)	31.9 (2.8)	31.0 (2.9)	30.9 (4.0)	23.7 (6.3)
FM, kg	8.9 (2.2)	9.5 (2.0)	9.9 (1.9)	9.9 (2.3)	13.6 (1.3)	13.9 (1.4)	13.9 (1.5)	14.3 (1.4)	17.1 (1.7)	17.2 (1.6)	17.6 (1.7)	18.0 (1.7)	22.1 (2.7)	22.3 (3.2)	23.0 (3.4)	26.2 (6.2)	16.2 (6.1)
FMI, kg/m^2^	3.3 [2.7, 3.8]	3.5 [2.9, 3.9]	3.5 [3.1, 3.9]	3.6 [3.0, 4.0]	5.0 [4.6, 5.2]	5.0 [4.6, 5.3]	4.9 [4.6, 5.2]	5.0 [4.6, 5.3]	6.2 [5.9, 6.6]	6.2 [5.9, 6.6]	6.2 [5.9, 6.6]	6.3 [5.9, 6.7]	8.2 [7.6, 8.9]	7.8 [7.4, 8.8]	8.0 [7.5, 8.8]	8.6 [7.7, 9.9]	5.5 [4.3, 7.1]
FFM, kg	45.1 (4.2)	49.4 (3.8)	52.9 (4.3)	57.6 (5.3)	44.8 (3.9)	49.3 (3.8)	51.8 (3.9)	56.7 (4.7)	44.5 (3.7)	48.2 (3.5)	51.7 (3.6)	57.0 (5.1)	43.0 (3.7)	47.5 (3.5)	50.9 (3.6)	58.0 (5.7)	50.6 (6.3)
FFMI, kg/m^2^	16.1 [15.6, 16.6]	17.5 [17.2, 17.7]	18.3 [18.1, 18.6]	19.4 [19.1, 20.0]	16.4 [15.9, 16.7]	17.5 [17.2, 17.7]	18.4 [18.2, 18.6]	19.5 [19.2, 20.0]	16.4 [16.0, 16.7]	17.5 [17.2, 17.7]	18.4 [18.2, 18.6]	19.6 [19.3, 20.2]	16.3 [15.8, 16.7]	17.5 [17.3, 17.7]	18.4 [18.2, 18.6]	19.9 [19.4, 20.7]	18.0 [17.0, 18.9]

FEV_1_, L	3.0 (0.7)	3.2 (0.7)	3.3 (0.6)	3.6 (0.7)	2.8 (0.6)	3.0 (0.6)	3.1 (0.6)	3.4 (0.6)	2.6 (0.6)	2.8 (0.6)	3.0 (0.6)	3.2 (0.6)	2.3 (0.6)	2.6 (0.6)	2.8 (0.6)	3.1 (0.6)	3.0 (0.7)
FVC, L	3.7 (0.7)	4.0 (0.7)	4.2 (0.7)	4.5 (0.7)	3.5 (0.7)	3.8 (0.6)	3.9 (0.7)	4.2 (0.7)	3.4 (0.6)	3.6 (0.7)	3.8 (0.6)	4.0 (0.6)	3.0 (0.6)	3.4 (0.6)	3.5 (0.7)	3.9 (0.7)	3.8 (0.7)
VC, %	98.6 (12.7)	103.5 (12.9)	106.1 (12.4)	108.3 (11.9)	99.7 (14.5)	104.2 (12.5)	105.2 (12.6)	107.8 (12.4)	97.8 (13.1)	101.8 (12.7)	103.1 (13.1)	103.3 (12.4)	94.8 (15.7)	99.7 (13.8)	99.7 (14.3)	100.2 (12.7)	101.9 (13.4)
Restrictive ventilatory impairment, %	24 (6.5)	3 (1.2)	3 (1.6)	0 (0.0)	17 (7.2)	4 (1.4)	1 (0.4)	1 (0.6)	15 (7.4)	11 (5.1)	11 (4.0)	6 (2.5)	20 (16.8)	12 (6.5)	14 (6.0)	21 (5.3)	163 (4.4)
FEV_1_/FVC, %	79.3 (9.4)	79.8 (7.7)	78.8 (7.2)	80.4 (5.7)	77.8 (7.5)	78.2 (6.9)	78.7 (5.9)	79.9 (5.5)	77.4 (8.3)	78.0 (7.4)	78.9 (5.5)	80.1 (5.7)	77.1 (9.0)	78.3 (6.5)	79.3 (6.1)	80.8 (5.7)	79.0 (7.1)
Obstructive ventilatory impairment, %	44 (11.9)	28 (11.4)	19 (10.1)	5 (3.8)	22 (9.4)	31 (10.6)	17 (7.0)	8 (4.9)	26 (12.8)	24 (11.1)	19 (7.0)	13 (5.4)	19 (16.0)	11 (6.0)	17 (7.3)	11 (2.8)	314 (8.4)

Leisure time physical activity, MET-min/week	87.9 [8.5, 259.5]	129.6 [17.5, 251.2]	124.7 [8.8, 306.0]	128.3 [29.7, 315.0]	108.0 [24.8, 288.6]	130.0 [18.0, 261.0]	105.0 [18.0, 278.0]	84.9 [9.0, 253.5]	99.2 [26.7, 231.1]	99.9 [16.8, 235.9]	85.7 [18.0, 246.9]	90.0 [9.0, 238.5]	78.0 [16.2, 184.6]	113.4 [20.4, 261.8]	65.6 [11.8, 180.0]	43.9 [0.0, 157.5]	90.0 [12.4, 248.4]

Education level, %																	
Below high school	197 (53.2)	128 (52.0)	103 (54.8)	71 (54.6)	127 (54.0)	164 (56.0)	135 (55.6)	76 (46.6)	132 (65.0)	118 (54.4)	150 (55.1)	133 (55.0)	83 (69.7)	114 (62.0)	138 (59.2)	208 (52.3)	2,077 (55.6)
Vocational school, junior college, or technical college	56 (15.1)	31 (12.6)	20 (10.6)	13 (10.0)	25 (10.6)	42 (14.3)	26 (10.7)	22 (13.5)	19 (9.4)	23 (10.6)	24 (8.8)	40 (16.5)	7 (5.9)	16 (8.7)	26 (11.2)	66 (16.6)	456 (12.2)
University or graduate school	113 (30.5)	79 (32.1)	62 (33.0)	44 (33.8)	79 (33.6)	86 (29.4)	81 (33.3)	62 (38.0)	49 (24.1)	62 (28.6)	94 (34.6)	67 (27.7)	26 (21.8)	49 (26.6)	62 (26.6)	117 (29.4)	1,132 (30.3)
Other	4 (1.1)	5 (2.0)	0 (0.0)	0 (0.0)	2 (0.9)	0 (0.0)	0 (0.0)	0 (0.0)	0 (0.0)	1 (0.5)	2 (0.7)	0 (0.0)	0 (0.0)	1 (0.5)	3 (1.3)	1 (0.3)	19 (0.5)
Unknown	0 (0.0)	3 (1.2)	3 (1.6)	2 (1.5)	2 (0.9)	1 (0.3)	1 (0.4)	3 (1.8)	3 (1.5)	13 (6.0)	2 (0.7)	2 (0.8)	3 (2.5)	4 (2.2)	4 (1.7)	6 (1.5)	52 (1.4)

Smoking status, %																	
Never smoker	109 (29.5)	76 (30.9)	56 (29.8)	37 (28.5)	79 (33.6)	95 (32.4)	74 (30.5)	43 (26.4)	59 (29.1)	64 (29.5)	67 (24.6)	64 (26.4)	42 (35.3)	50 (27.2)	66 (28.3)	95 (23.9)	1,076 (28.8)
Ex-smoker	181 (48.9)	114 (46.3)	90 (47.9)	54 (41.5)	108 (46.0)	142 (48.5)	123 (50.6)	83 (50.9)	104 (51.2)	105 (48.4)	156 (57.4)	112 (46.3)	63 (52.9)	103 (56.0)	131 (56.2)	202 (50.8)	1,871 (50.1)
1–19 cigarettes/day	47 (12.7)	27 (11.0)	22 (11.7)	18 (13.8)	23 (9.8)	28 (9.6)	25 (10.3)	16 (9.8)	19 (9.4)	22 (10.1)	19 (7.0)	26 (10.7)	8 (6.7)	17 (9.2)	16 (6.9)	47 (11.8)	380 (10.2)
≥20 cigarettes/day	33 (8.9)	28 (11.4)	18 (9.6)	20 (15.4)	22 (9.4)	26 (8.9)	21 (8.6)	18 (11.0)	18 (8.9)	20 (9.2)	27 (9.9)	39 (16.1)	3 (2.5)	12 (6.5)	18 (7.7)	51 (12.8)	374 (10.0)
Unknown	0 (0.0)	1 (0.4)	2 (1.1)	1 (0.8)	3 (1.3)	2 (0.7)	0 (0.0)	3 (1.8)	3 (1.5)	6 (2.8)	3 (1.1)	1 (0.4)	3 (2.5)	2 (1.1)	2 (0.9)	3 (0.8)	35 (0.9)
Passive smoking, %	83 (22.4)	64 (26.0)	55 (29.3)	36 (27.7)	42 (17.9)	58 (19.8)	56 (23.0)	44 (27.0)	22 (10.8)	42 (19.4)	67 (24.6)	71 (29.3)	13 (10.9)	26 (14.1)	51 (21.9)	134 (33.7)	864 (23.1)

Drinking status, %																	
Never drinker	66 (17.8)	41 (16.7)	23 (12.2)	25 (19.2)	53 (22.6)	39 (13.3)	45 (18.5)	17 (10.4)	43 (21.2)	34 (15.7)	43 (15.8)	39 (16.1)	37 (31.1)	31 (16.8)	41 (17.6)	89 (22.4)	666 (17.8)
Ex-drinker	16 (4.3)	13 (5.3)	5 (2.7)	6 (4.6)	8 (3.4)	9 (3.1)	8 (3.3)	8 (4.9)	11 (5.4)	10 (4.6)	7 (2.6)	6 (2.5)	4 (3.4)	9 (4.9)	10 (4.3)	14 (3.5)	144 (3.9)
<23 g	157 (42.4)	106 (43.1)	84 (44.7)	42 (32.3)	95 (40.4)	121 (41.3)	93 (38.3)	60 (36.8)	68 (33.5)	81 (37.3)	105 (38.6)	90 (37.2)	39 (32.8)	68 (37.0)	76 (32.6)	149 (37.4)	1,434 (38.4)
≥23 g	131 (35.4)	85 (34.6)	74 (39.4)	56 (43.1)	77 (32.8)	123 (42.0)	97 (39.9)	78 (47.9)	79 (38.9)	86 (39.6)	115 (42.3)	106 (43.8)	37 (31.1)	75 (40.8)	104 (44.6)	145 (36.4)	1,468 (39.3)
Unknown	0 (0.0)	1 (0.4)	2 (1.1)	1 (0.8)	2 (0.9)	1 (0.3)	0 (0.0)	0 (0.0)	2 (1.0)	6 (2.8)	2 (0.7)	1 (0.4)	2 (1.7)	1 (0.5)	2 (0.9)	1 (0.3)	24 (0.6)
History of disease, %																	
Asthma	15 (4.1)	12 (4.9)	14 (7.4)	4 (3.1)	15 (6.4)	19 (6.5)	17 (7.0)	8 (4.9)	11 (5.4)	9 (4.1)	19 (7.0)	15 (6.2)	13 (10.9)	12 (6.5)	12 (5.2)	31 (7.8)	226 (6.0)
Chronic bronchitis	0 (0.0)	4 (1.6)	5 (2.7)	2 (1.5)	2 (0.9)	2 (0.7)	0 (0.0)	0 (0.0)	1 (0.5)	1 (0.5)	2 (0.7)	1 (0.4)	1 (0.8)	2 (1.1)	1 (0.4)	2 (0.5)	26 (0.7)
COPD	1 (0.3)	0 (0.0)	4 (2.1)	0 (0.0)	2 (0.9)	0 (0.0)	1 (0.4)	0 (0.0)	0 (0.0)	1 (0.5)	2 (0.7)	2 (0.8)	3 (2.5)	1 (0.5)	0 (0.0)	1 (0.3)	18 (0.5)

BF%, body fat percentage; BMI, body mass index; COPD, chronic obstructive pulmonary disease; FEV_1_, forced expiratory volume at 1 s; FFM, fat-free mass; FFMI, fat-free mass index; FM, fat mass; FMI, fat mass index; FVC, forced vital capacity; METs, metabolic equivalents; Q, quartile, VC vital capacity.

Values are expressed as means (standard deviations) or medians (interquartile ranges) for continuous variables, or numbers (%) for categorical variables.

The FMI was categorized into the following quartile cutoff points: Q1, <4.3; Q2, 4.3–5.4; Q3, 5.5–7.1; and Q4, ≥7.1 kg/m^2^.

The FFMI was categorized into the following quartile cutoff points: Q1, <17.0; Q2, 17.0–17.9; Q3, 18.0–18.9; and Q4, ≥18.9 kg/m^2^.

Restrictive ventilatory impairment was defined as reduced VC of <80% of the predicted value. Obstructive ventilatory impairment was defined as a reduction in FEV_1_ indicated by FVC <70%.

**Table 2.** Characteristics of combined FMI and FFMI among women

FMI	Q1				Q2				Q3				Q4				All participants

FFMI	Q1	Q2	Q3	Q4	Q1	Q2	Q3	Q4	Q1	Q2	Q3	Q4	Q1	Q2	Q3	Q4	
Number	892	645	441	228	676	637	541	350	505	584	639	477	133	336	586	1,151	8,821
Age, years	53.0 (13.9)	51.1 (13.6)	50.9 (13.5)	51.2 (12.3)	56.2 (14.0)	55.4 (13.5)	54.7 (13.5)	54.4 (13.9)	59.3 (11.9)	59.6 (11.6)	58.9 (12.5)	56.3 (13.3)	63.6 (11.8)	62.7 (10.7)	60.4 (11.8)	56.4 (12.9)	56.2 (13.4)
Height, cm	157.3 (5.4)	157.6 (5.6)	157.2 (5.4)	158.3 (5.5)	155.9 (5.6)	156.0 (5.6)	156.5 (5.7)	156.5 (5.9)	154.6 (5.4)	154.7 (5.7)	155.3 (5.6)	156.1 (5.9)	152.8 (5.6)	153.2 (5.9)	153.6 (5.4)	155.2 (5.9)	155.8 (5.8)
BMI, kg/m^2^	17.7 (1.0)	18.9 (0.8)	19.7 (0.8)	20.8 (0.9)	19.8 (0.7)	20.7 (0.5)	21.5 (0.5)	22.5 (0.7)	21.4 (0.7)	22.4 (0.6)	23.2 (0.6)	24.3 (0.8)	23.5 (1.0)	24.8 (1.2)	25.9 (1.6)	28.4 (2.8)	22.3 (3.5)
BF%, %	22.1 (3.8)	21.6 (3.3)	21.0 (3.1)	20.5 (3.0)	29.8 (1.8)	28.4 (1.6)	27.7 (1.5)	26.4 (1.6)	35.0 (1.8)	33.8 (1.6)	32.9 (1.6)	31.6 (1.5)	40.5 (2.3)	40.0 (2.7)	39.6 (3.2)	39.6 (4.1)	30.7 (7.3)
FM, kg	9.8 (2.1)	10.2 (2.0)	10.3 (1.9)	10.7 (1.9)	14.3 (1.4)	14.4 (1.4)	14.6 (1.5)	14.6 (1.6)	17.9 (1.7)	18.1 (1.8)	18.5 (1.9)	18.8 (1.9)	22.2 (2.5)	23.4 (3.2)	24.3 (3.7)	27.3 (5.8)	17.1 (6.5)
FMI, kg/m^2^	4.1 [3.4, 4.6]	4.3 [3.6, 4.8]	4.3 [3.7, 4.8]	4.5 [3.9, 4.9]	5.9 [5.5, 6.3]	5.9 [5.5, 6.3]	6.0 [5.6, 6.3]	6.0 [5.5, 6.4]	7.4 [7.1, 7.9]	7.5 [7.1, 8.0]	7.7 [7.2, 8.1]	7.7 [7.3, 8.1]	9.2 [8.8, 9.8]	9.7 [9.1, 10.5]	9.9 [9.2, 11.0]	10.7 [9.6, 12.4]	6.7 [5.1, 8.6]
FFM, kg	34.2 (2.7)	36.8 (2.6)	38.5 (2.7)	41.4 (3.0)	33.8 (2.7)	36.1 (2.7)	38.1 (2.8)	40.6 (3.4)	33.2 (2.7)	35.5 (2.7)	37.6 (2.7)	40.5 (3.6)	32.7 (2.6)	34.9 (2.7)	36.9 (2.7)	41.2 (4.1)	37.1 (4.0)
FFMI, kg/m^2^	13.9 [13.5, 14.2]	14.8 [14.6, 15.0]	15.5 [15.3, 15.7]	16.3 [16.1, 16.7]	14.0 [13.6, 14.2]	14.8 [14.6, 15.0]	15.5 [15.4, 15.7]	16.4 [16.2, 16.8]	14.0 [13.6, 14.2]	14.8 [14.6, 15.0]	15.5 [15.4, 15.7]	16.4 [16.2, 16.8]	14.1 [13.8, 14.3]	14.9 [14.7, 15.1]	15.6 [15.4, 15.8]	16.8 [16.4, 17.5]	15.2 [14.5, 16.0]

FEV_1_, L	2.3 (0.5)	2.4 (0.5)	2.5 (0.5)	2.6 (0.5)	2.2 (0.5)	2.3 (0.5)	2.4 (0.5)	2.5 (0.5)	2.1 (0.4)	2.2 (0.4)	2.3 (0.4)	2.4 (0.5)	2.0 (0.4)	2.0 (0.4)	2.1 (0.4)	2.3 (0.5)	2.3 (0.5)
FVC, L	2.8 (0.5)	3.0 (0.5)	3.1 (0.5)	3.2 (0.5)	2.7 (0.5)	2.8 (0.5)	2.9 (0.5)	3.0 (0.5)	2.6 (0.5)	2.7 (0.5)	2.8 (0.5)	2.9 (0.5)	2.4 (0.5)	2.5 (0.5)	2.6 (0.5)	2.8 (0.5)	2.8 (0.5)
VC, %	97.5 (13.0)	101.5 (12.5)	105.9 (13.7)	109.4 (13.2)	98.8 (13.1)	103.0 (13.5)	106.3 (12.7)	108.7 (13.9)	99.9 (13.7)	103.7 (13.0)	106.3 (13.2)	108.4 (13.9)	99.6 (15.0)	101.1 (13.5)	102.4 (12.8)	103.6 (12.9)	103.1 (13.6)
Restrictive ventilatory impairment, %	58 (6.5)	18 (2.8)	4 (0.9)	4 (1.8)	44 (6.5)	23 (3.6)	2 (0.4)	6 (1.7)	33 (6.5)	12 (2.1)	9 (1.4)	3 (0.6)	9 (6.8)	17 (5.1)	17 (2.9)	27 (2.3)	286 (3.2)
FEV_1_/FVC, %	82.3 (7.5)	81.9 (6.8)	81.7 (6.1)	81.1 (5.4)	81.1 (6.3)	81.5 (5.9)	80.9 (5.4)	80.9 (5.6)	80.4 (5.9)	80.3 (5.4)	81.1 (5.0)	81.2 (5.2)	80.9 (5.8)	81.0 (5.0)	81.6 (5.1)	82.1 (4.8)	81.4 (5.8)
Obstructive ventilatory impairment, %	35 (3.9)	24 (3.7)	18 (4.1)	8 (3.5)	25 (3.7)	16 (2.5)	11 (2.0)	11 (3.1)	21 (4.2)	20 (3.4)	8 (1.3)	12 (2.5)	6 (4.5)	7 (2.1)	13 (2.2)	14 (1.2)	249 (2.8)

Leisure time physical activity, MET-min/week	57.9 [4.0, 169.9]	63.0 [9.0, 192.9]	67.5 [16.8, 214.7]	92.0 [17.9, 270.0]	58.9 [8.4, 160.2]	66.9 [8.4, 207.6]	81.0 [9.0, 210.0]	90.0 [16.8, 250.9]	57.9 [8.4, 159.4]	90.0 [18.0, 195.6]	84.9 [10.8, 228.2]	76.5 [8.4, 218.6]	60.7 [12.9, 159.4]	67.7 [9.0, 190.3]	63.0 [8.4, 173.6]	54.0 [0.0, 162.0]	66.0 [8.4, 192.9]
Education level %																	
Below high school	443 (49.7)	296 (45.9)	190 (43.1)	118 (51.8)	370 (54.7)	342 (53.7)	297 (54.9)	191 (54.6)	302 (59.8)	343 (58.7)	355 (55.6)	270 (56.6)	77 (57.9)	226 (67.3)	371 (63.3)	656 (57.0)	4,847 (54.9)
Vocational school, junior college, or technical college	290 (32.5)	220 (34.1)	171 (38.8)	77 (33.8)	215 (31.8)	205 (32.2)	175 (32.3)	112 (32.0)	150 (29.7)	195 (33.4)	207 (32.4)	144 (30.2)	40 (30.1)	90 (26.8)	166 (28.3)	393 (34.1)	2,850 (32.3)
University or graduate school	142 (15.9)	122 (18.9)	74 (16.8)	32 (14.0)	84 (12.4)	85 (13.3)	66 (12.2)	40 (11.4)	46 (9.1)	38 (6.5)	71 (11.1)	56 (11.7)	12 (9.0)	14 (4.2)	38 (6.5)	85 (7.4)	1,005 (11.4)
Other	3 (0.3)	2 (0.3)	0 (0.0)	1 (0.4)	2 (0.3)	1 (0.2)	2 (0.4)	2 (0.6)	3 (0.6)	3 (0.5)	1 (0.2)	1 (0.2)	0 (0.0)	2 (0.6)	3 (0.5)	4 (0.3)	30 (0.3)
Unknown	14 (1.6)	5 (0.8)	6 (1.4)	0 (0.0)	5 (0.7)	4 (0.6)	1 (0.2)	5 (1.4)	4 (0.8)	5 (0.9)	5 (0.8)	6 (1.3)	4 (3.0)	4 (1.2)	8 (1.4)	13 (1.1)	89 (1.0)

Smoking status, %																	
Never smoker	698 (78.3)	495 (76.7)	320 (72.6)	163 (71.5)	540 (79.9)	520 (81.6)	439 (81.1)	260 (74.3)	400 (79.2)	472 (80.8)	523 (81.8)	360 (75.5)	111 (83.5)	277 (82.4)	476 (81.2)	870 (75.6)	6,924 (78.5)
Ex-smoker	110 (12.3)	92 (14.3)	76 (17.2)	41 (18.0)	86 (12.7)	77 (12.1)	75 (13.9)	56 (16.0)	68 (13.5)	73 (12.5)	79 (12.4)	72 (15.1)	15 (11.3)	41 (12.2)	69 (11.8)	177 (15.4)	1,207 (13.7)
1–19 cigarettes/day	61 (6.8)	39 (6.0)	36 (8.2)	19 (8.3)	31 (4.6)	31 (4.9)	19 (3.5)	25 (7.1)	33 (6.5)	27 (4.6)	29 (4.5)	31 (6.5)	4 (3.0)	8 (2.4)	27 (4.6)	62 (5.4)	482 (5.5)
≥20 cigarettes/day	15 (1.7)	13 (2.0)	5 (1.1)	3 (1.3)	15 (2.2)	4 (0.6)	8 (1.5)	8 (2.3)	2 (0.4)	5 (0.9)	5 (0.8)	6 (1.3)	1 (0.8)	7 (2.1)	9 (1.5)	33 (2.9)	139 (1.6)
Unknown	8 (0.9)	6 (0.9)	4 (0.9)	2 (0.9)	4 (0.6)	5 (0.8)	0 (0.0)	1 (0.3)	2 (0.4)	7 (1.2)	3 (0.5)	8 (1.7)	2 (1.5)	3 (0.9)	5 (0.9)	9 (0.8)	69 (0.8)
Passive smoking, %	162 (18.2)	116 (18.0)	86 (19.5)	52 (22.8)	113 (16.7)	108 (17.0)	91 (16.8)	74 (21.1)	78 (15.4)	124 (21.2)	112 (17.5)	96 (20.1)	19 (14.3)	52 (15.5)	110 (18.8)	251 (21.8)	1,644 (18.6)

Drinking status, %																	
Never drinker	448 (50.2)	280 (43.4)	207 (46.9)	92 (40.4)	390 (57.7)	326 (51.2)	254 (47.0)	158 (45.1)	292 (57.8)	327 (56.0)	325 (50.9)	231 (48.4)	95 (71.4)	203 (60.4)	322 (54.9)	622 (54.0)	4,572 (51.8)
Ex-drinker	21 (2.4)	17 (2.6)	8 (1.8)	5 (2.2)	8 (1.2)	12 (1.9)	13 (2.4)	10 (2.9)	7 (1.4)	9 (1.5)	7 (1.1)	9 (1.9)	4 (3.0)	4 (1.2)	6 (1.0)	24 (2.1)	164 (1.9)
<23 g	340 (38.1)	294 (45.6)	168 (38.1)	94 (41.2)	228 (33.7)	250 (39.2)	220 (40.7)	143 (40.9)	171 (33.9)	213 (36.5)	247 (38.7)	189 (39.6)	28 (21.1)	105 (31.2)	211 (36.0)	405 (35.2)	3,306 (37.5)
≥23 g	79 (8.9)	53 (8.2)	55 (12.5)	37 (16.2)	47 (7.0)	45 (7.1)	54 (10.0)	38 (10.9)	35 (6.9)	32 (5.5)	56 (8.8)	42 (8.8)	4 (3.0)	21 (6.2)	43 (7.3)	96 (8.3)	737 (8.4)
Unknown	4 (0.4)	1 (0.2)	3 (0.7)	0 (0.0)	3 (0.4)	4 (0.6)	0 (0.0)	1 (0.3)	0 (0.0)	3 (0.5)	4 (0.6)	6 (1.3)	2 (1.5)	3 (0.9)	4 (0.7)	4 (0.3)	42 (0.5)

History of disease, %																	
Asthma	74 (8.3)	48 (7.4)	30 (6.8)	8 (3.5)	43 (6.4)	36 (5.7)	32 (5.9)	22 (6.3)	27 (5.3)	26 (4.5)	35 (5.5)	25 (5.2)	5 (3.8)	18 (5.4)	49 (8.4)	113 (9.8)	591 (6.7)
Chronic bronchitis	8 (0.9)	6 (0.9)	3 (0.7)	2 (0.9)	5 (0.7)	8 (1.3)	5 (0.9)	2 (0.6)	3 (0.6)	5 (0.9)	2 (0.3)	5 (1.0)	2 (1.5)	3 (0.9)	8 (1.4)	17 (1.5)	84 (1.0)
COPD	1 (0.1)	0 (0.0)	1 (0.2)	0 (0.0)	0 (0.0)	1 (0.2)	0 (0.0)	1 (0.3)	2 (0.4)	1 (0.2)	0 (0.0)	0 (0.0)	0 (0.0)	1 (0.3)	0 (0.0)	1 (0.1)	9 (0.1)

BF%, body fat percentage; BMI, body mass index; COPD, chronic obstructive pulmonary disease; FEV_1_, forced expiratory volume at 1 s; FFM, fat-free mass; FFMI, fat-free mass index; FM, fat mass; FMI, fat mass index; FVC, forced vital capacity; METs, metabolic equivalents; Q, quartile, VC, vital capacity.

Values are expressed as means (standard deviations) or medians (interquartile ranges) for continuous variables, or numbers (%) for categorical variables.

The FMI was categorized into the following quartile cutoff points: Q1, <5.1; Q2, 5.1–6.6; Q3, 6.7–8.6; and Q4, ≥8.6 kg/m^2^.

The FFMI was categorized into the following quartile cutoff points: Q1, <14.5; Q2, 14.5–15.1; Q3, 15.2–16.0; and Q4, ≥16.0 kg/m^2^.

Restrictive ventilatory impairment was defined as reduced VC of <80% of the predicted value.

Obstructive ventilatory impairment was defined as a reduction in FEV_1_ indicated by FVC of <70%.

FMI was associated with lower FEV_1_ after adjustments for several potential confounders (*P* for linear trend <0.001). Similarly, the FMI was associated with lower FVC after adjustments for several potential confounders in both men and women (*P* for linear trend <0.001) (eTable 3). Conversely, higher FFMI was associated with higher FEV_1_ and FVC in both men and women (*P* for linear trend <0.001) (eTable 4).

The association of combined FMI and FFMI with FEV_1_ is shown in Table 3. FMI was inversely associated with FEV_1_ in all FFMI subgroups, whereas FFMI was positively associated with FEV_1_ in all FMI subgroups. Similarly, a higher FMI was associated with a lower FVC in all FFMI subgroups, and a higher FFMI was associated with a higher FVC in all FMI subgroups (Table 4).

**Table 3.** Association of combined FMI and FFMI with FEV_1_

Men	FMI Q1			FMI Q2			FMI Q3			FMI Q4			Multiple linear regression of FFMI subgroups^b^		

	LS means FEV_1_ (L), 95% CI												β	SE	*P*
FFMI Q1	2.93	(2.86–3.00)		2.84	(2.76–2.93)		2.79	(2.70–2.87)		2.62	(2.52–2.71)		−0.06	0.01	<0.001
FFMI Q2	3.06	(2.99–3.14)		3.00	(2.92–3.07)		2.86	(2.78–2.94)		2.79	(2.70–2.88)		−0.07	0.01	<0.001
FFMI Q3	3.07	(2.99–3.16)		3.00	(2.92–3.08)		2.97	(2.90–3.05)		2.85	(2.77–2.93)		−0.04	0.01	<0.001
FFMI Q4	3.25	(3.15–3.34)		3.17	(3.08–3.26)		3.04	(2.96–3.12)		2.95	(2.88–3.02)		−0.05	0.01	<0.001
Multiple linear regression of FMI subgroups^a^	β	SE	*P*	β	SE	*P*	β	SE	*P*	β	SE	*P*			
	0.08	0.01	<0.001	0.10	0.01	<0.001	0.08	0.01	<0.001	0.06	0.01	<0.001			

Women	LS means FEV_1_ (L), 95% CI												Multiple linear regression of FFMI subgroups^b^		

FFMI Q1	2.22	(2.18–2.26)		2.18	(2.14–2.22)		2.15	(2.11–2.20)		2.13	(2.06–2.19)		−0.02	0.003	<0.001
FFMI Q2	2.30	(2.26–2.34)		2.28	(2.24–2.32)		2.24	(2.20–2.29)		2.17	(2.12–2.21)		−0.02	0.003	<0.001
FFMI Q3	2.37	(2.32–2.41)		2.33	(2.29–2.38)		2.32	(2.27–2.36)		2.21	(2.17–2.26)		−0.03	0.003	<0.001
FFMI Q4	2.45	(2.40–2.51)		2.40	(2.35–2.44)		2.36	(2.31–2.40)		2.27	(2.23–2.31)		−0.02	0.002	<0.001
Multiple linear regression of FMI subgroups^a^	β	SE	*P*	β	SE	*P*	β	SE	*P*	β	SE	*P*			
	0.09	0.01	<0.001	0.08	0.01	<0.001	0.08	0.01	<0.001	0.04	0.01	<0.001			

CI, confidence interval; FEV_1_, forced expiratory volume at 1 s; FFMI, fat-free mass index; FMI, fat mass index; LS, least squares; Q, quartile.

Adjusted for age, education level (below high school; vocational school, junior college, or technical college; university or graduate school; other; unknown), smoking status (never smoker, ex-smoker, current smoker [1–19 or ≥20 cigarettes/day], unknown), passive smoking (yes, no), and drinking status (never drinker, ex-drinker, current drinker [<23 or ≥23 g/day], unknown).

^a^Multiple regression analysis with stratified FMI and actual FFMI as continuous variables.

^b^Multiple regression analysis with stratified FFMI and actual FMI as continuous variables.

**Table 4.** Association of combined FMI and FFMI with FVC

Men	FMI Q1			FMI Q2			FMI Q3			FMI Q4			Multiple linear regression of FFMI subgroups^b^		

	LS means FVC (L), 95% CI												β	SE	*P*
FFMI Q1	3.72	(3.64–3.81)		3.64	(3.55–3.74)		3.56	(3.46–3.66)		3.33	(3.21–3.45)		−0.08	0.01	<0.001
FFMI Q2	3.89	(3.80–3.98)		3.86	(3.77–3.95)		3.66	(3.57–3.76)		3.53	(3.43–3.63)		−0.08	0.01	<0.001
FFMI Q3	3.98	(3.88–4.08)		3.86	(3.77–3.96)		3.79	(3.69–3.88)		3.60	(3.50–3.69)		−0.07	0.01	<0.001
FFMI Q4	4.15	(4.04–4.27)		4.05	(3.94–4.15)		3.85	(3.76–3.95)		3.72	(3.63–3.80)		−0.06	0.01	<0.001
Multiple linear regression of FMI subgroups^a^	β	SE	*P*	β	SE	*P*	β	SE	*P*	β	SE	*P*			
	0.12	0.01	<0.001	0.12	0.01	<0.001	0.08	0.01	<0.001	0.06	0.01	<0.001			

Women	LS means FVC (L), 95% CI												Multiple linear regression of FFMI subgroups^b^		

FFMI Q1	2.74	(2.69–2.79)		2.71	(2.66–2.76)		2.69	(2.63–2.74)		2.61	(2.53–2.69)		−0.02	0.004	<0.001
FFMI Q2	2.86	(2.81–2.91)		2.83	(2.78–2.88)		2.79	(2.74–2.85)		2.66	(2.60–2.72)		−0.03	0.004	<0.001
FFMI Q3	2.96	(2.90–3.01)		2.92	(2.87–2.98)		2.87	(2.82–2.92)		2.71	(2.66–2.76)		−0.04	0.004	<0.001
FFMI Q4	3.08	(3.02–3.15)		2.99	(2.94–3.05)		2.92	(2.87–2.98)		2.79	(2.74–2.83)		−0.03	0.003	<0.001
Multiple linear regression of FMI subgroups^a^	β	SE	*P*	β	SE	*P*	β	SE	*P*	β	SE	*P*			
	0.13	0.001	<0.001	0.11	0.001	<0.001	0.09	0.001	<0.001	0.04	0.001	<0.001			

CI, confidence interval; FFMI, fat-free mass index; FMI, fat mass index; FVC, forced vital capacity; LS, least squares; Q, quartile.

Adjusted for age, education level (below high school; vocational school, junior college, or technical college; university or graduate school; other; unknown), smoking status (never smoker, ex-smoker, current smoker [1–19 or ≥20 cigarettes/day], unknown), passive smoking (yes, no), and drinking status (never drinker, ex-drinker, current drinker [<23 or ≥23 g/day], unknown).

^a^Multiple regression analysis with stratified FMI and actual FFMI as continuous variables.

^b^Multiple regression analysis with stratified FFMI and actual FMI as continuous variables.

We conducted several sensitivity analyses to test our findings. First, when we restricted our study to never smokers, the results were substantially unchanged compared with those using all participants (eTable 5 for FEV_1_; eTable 6 for FVC). Second, to examine the effect of age on the association between body composition and lung function, we conducted a stratified analysis according to age tertile (men: <58 years, 58–67 years, ≥67 years and women: <52 years, 52–64 years, ≥64 years). Even in the stratified subgroups, the findings were essentially unchanged (eTable 7, eTable 8, eTable 9, eTable 10, eTable 11, and eTable 12). Finally, even when we restricted the analysis to participants without respiratory disease, the results were substantially unchanged compared with those using all participants (eTable 13 and eTable 14).

## DISCUSSION

In the present study, we found that FMI was inversely associated with lung function variables, such as FEV_1_ and FVC, in all FFMI subgroups, whereas FFMI was positively associated with lung function in all FMI subgroups. These findings suggest that FMI is associated with lower lung function independent of FFMI, whereas FFMI is associated with higher lung function independent of FMI.

Many studies have examined the association between body composition and lung function.^10^^–^^21^ The findings of a study on 7,801 Taiwanese individuals showed that BF% was inversely associated with FEV_1_ and FVC, even among patients with normal weight determined by BMI (18.5–24.9 kg/m).^15^ In a study of 1,746 Brazilian participants, FM was reportedly inversely associated with FEV_1_ and FVC.^18^ Conversely, regarding the association between FFM and lung function, in the Avon Longitudinal Study of Parents and Children, a birth cohort study, higher FFMI was associated with higher levels of FEV_1_ and FVC.^13^ Skeletal muscle mass, which is the main component of FFM, was positively associated with FEV_1_ and FVC in 240,556 Korean adults without known lung disease.^29^ These studies suggest that FM and FFM may have different roles in lung function. However, although it might be necessary to consider both FM and FFM to clarify the association between body composition and lung function, many studies did not consider both FM and FFM.^10^^–^^12^^,^^14^^,^^15^^,^^18^^–^^21^ Previous studies have mutually adjusted both FM and FFM to consider body composition.^13^^,^^16^^–^^18^^,^^34^ However, because FM and FFM are highly correlated, a model that considers both FM and FFM simultaneously is not ideal owing to collinearity. Therefore, we combined FMI and FFMI to avoid multicollinearity, and examined the association between FFMI and lung function in FMI subgroups and the association between FMI and lung function in FFMI subgroups. As a result, we observed that a higher FMI was associated with lower lung function in all FFMI subgroups, whereas a higher FFMI was associated with higher lung function in all FMI subgroups among a Japanese population, which is consistent with previous studies.^10^^–^^21^^,^^29^

We raised some possibilities of confounding and performed stratified analysis. First, smoking may have been an important confounding factor, since smoking is strongly associated with reduced lung function and increased FM.^30^^,^^31^ Although we limited our analysis to never-smokers, FMI was inversely associated with lung function and FFMI was positively associated with lung function. Second, since respiratory disease is associated with higher FM and lower FFM,^32^^,^^33^ we selected only participants without respiratory disease. Even after restricting to participants without respiratory disease, an inverse association between FMI and lung function and a positive association between FFMI and lung function were observed. Therefore, we considered that the association between body composition and lung function may be independent of smoking and respiratory disease.

Other potential mechanisms for the association between FFM and lung function have been considered. A higher FFM may reflect respiratory skeletal muscle mass and increase the ability to inflate and contract the lungs,^9^ resulting in greater FEV_1_ and FVC. Moreover, recent studies have suggested that skeletal muscle-derived humoral molecules such as growth differentiation factor-15 and irisin act protectively on lung constituent cells.^34^^–^^37^ Conversely, inflammation and oxidative stress promote skeletal muscle wasting and may be involved in the pathogenesis of decreased lung function.^38^^–^^40^ Therefore, greater FFMI may be associated with higher lung function and a protective factor for adequate lung function.

The accumulation of adipose tissue may be associated with reduced lung function via several mechanisms, such as mechanical effects on the thorax and inflammation. Accumulation of adipose tissue in the thoracic and abdominal regions may impede the expansion and excursion of the diaphragm and chest wall.^5^^,^^6^ These structural changes may also induce a reduction in lung compliance and mechanical impairment of respiratory muscles.^5^ Furthermore, excess adipose tissue produces several proinflammatory cytokines, such as tumor necrosis factor-α and interleukin-6, which may lead to reduced lung function.^5^^,^^6^ Therefore, higher FMI may be associated with decreased lung function.

This study has several strengths. First, this is the first study to show an association between combined FMI and FFMI and lung function. Second, since we used a relatively large population of 12,557 participants, we were able to stratify by sex and age and limit to never smokers or participants with no history of respiratory disease. Furthermore, since we classified 16 subgroups by combining FMI and FFMI, we were able to show the details of the associations with lung function due to differences in body composition.

However, our study had several limitations. First, lung function might have been measured with some error because lung function is dependent on the participant’s effort. Second, a measurement error in the BF% might have occurred because the BF% was measured using the bioelectric impedance method. However, a good correlation between whole-body FM measured using the bioelectric impedance method and whole-body FM measured by the dual-energy X-ray absorptiometry method has already been confirmed (men, *r* = 0.95; women, *r* = 0.92).^41^ Third, residual or unmeasured confounding may exist. We did not include physical activity and dietary habits as covariates. However, since the results of physical activity and dietary habits may appear as differences in body composition, we considered adjustment for these determinants of body composition might underestimate the association between body composition and lung function. Forth, this study enrolled only Japanese participants. Body composition and lung function vary according to race. Asians have higher body fat and lower FFM than that of people of other ethnicities.^42^^,^^43^ Furthermore, compared with Europeans, Asians have lower lung function parameters, such as FEV_1_ and FVC.^44^ Applying our results to other races is difficult; therefore, similar studies are required in other populations. Finally, this study used a cross-sectional design and could not definitively establish a causal relationship between combined FMI and FFMI and lung function. Prospective cohort studies are needed to clarify this causal relationship.

### Conclusion

This study demonstrated that FMI was inversely associated with lung function independent of FFMI. Conversely, FFMI was positively associated with lung function independent of FMI. This association was confirmed when stratified by age and restricted to participants who had never smoked. These findings suggest that reducing FMI and maintaining FFMI are important for improving respiratory health.

## SUPPLEMENTARY MATERIAL

eTable 1. Participant characteristics by FMI quartile

eTable 2. Participant characteristics by FFMI quartile

eTable 3. Adjusted least square means of lung function indices associated by FMI quartile group

eTable 4. Adjusted least square means of lung function indices according to FFMI quartile

eTable 5. Association of combined FMI and FFMI with FEV_1_ among never smokers

eTable 6. Association of combined FMI and FFMI with FVC among never smokers

eTable 7. Association of combined FMI and FFMI with FEV_1_ among the lowest age tertile

eTable 8. Association of combined FMI and FFMI with FEV_1_ among the second age tertile

eTable 9. Association of combined FMI and FFMI with FEV_1_ among the highest age tertile

eTable 10. Association of combined FMI and FFMI with FVC among the lowest age tertile

eTable 11. Association of combined FMI and FFMI with FVC among the second age tertile

eTable 12. Association of combined FMI and FFMI with FVC among the highest age tertile

eTable 13. Association of combined FMI and FFMI with FEV_1_ among participants without respiratory disease

eTable 14. Association of combined FMI and FFMI with FVC among participants without respiratory disease
