# Supplementary material for: Combined Fat Mass and Fat-free Mass Indices and Lung Function Among Japanese Population: The Tohoku Medical Megabank Community-based Cohort Study
Source: J Epidemiol. 2024 Mar 5;34(3):119–28. doi: 10.2188/jea.JE20220355 (PMC10853044; doi:10.2188/jea.JE20220355)
Supplement: Supplementary file 1 [file je-34-119-s001.pdf]

eTable 1. Participant characteristics by FMI quartile

FMI	Men	Q1 (<4.3)	Q2 (4.3–5.4)	Q3 (5.5–7.1)	Q4 (≥7.1)	*P* -value [a]	Women	Q1 (<5.1)	Q2 (5.1–6.6)	Q3 (6.7–8.6)	Q4 (≥8.6)	*P* -value [a]
Number	3,736	934	934	934	934		8,821	2,206	2,202	2,207	2,206	
Age, years	60.1 (14.0)	56.8 (15.5)	60.3 (13.5)	61.6 (12.7)	61.6 (13.7)	<0.001	56.2 (13.4)	56.1 (13.7)	56.4 (13.3)	56.7 (13.3)	55.5 (13.2)	0.034
Height, cm	167.5 (6.3)	168.7 (6.6)	167.6 (6.2)	167.1 (6.0)	166.7 (6.3)	<0.001	155.8 (5.8)	156.0 (5.6)	155.7 (5.9)	155.5 (5.7)	155.9 (5.9)	0.035
BMI, kg/m^2	23.8 (3.1)	20.7 (1.7)	22.7 (1.3)	24.3 (1.6)	27.4 (2.6)	<0.001	22.3 (3.5)	19.5 (1.9)	21.3 (2.1)	22.8 (2.4)	25.8 (3.6)	<0.001
BF%, %	23.7 (6.3)	15.9 (3.1)	21.8 (1.7)	25.7 (1.9)	31.5 (3.5)	<0.001	30.7 (7.3)	28.5 (6.6)	29.6 (6.8)	31.0 (7.0)	33.8 (7.5)	<0.001
FM, kg	16.2 (6.1)	9.4 (2.2)	13.9 (1.4)	17.5 (1.7)	24.1 (5.1)	<0.001	17.1 (6.5)	13.8 (4.3)	15.5 (4.9)	17.4 (5.6)	21.7 (7.6)	<0.001
FMI, kg/m^2	5.5 [4.3, 7.1]	3.4 [2.8, 3.9]	5.0 [4.6, 5.2]	6.2 [5.9, 6.6]	8.2 [7.5, 9.2]	<0.001	6.7 [5.1, 8.6]	5.6 [4.4, 7.0]	6.2 [4.9, 7.8]	7.0 [5.5, 8.7]	8.8 [6.6, 10.8]	<0.001
FFM, kg	50.6 (6.3)	49.5 (6.1)	50.1 (5.6)	50.7 (6.0)	52.2 (7.2)	<0.001	37.1 (4.0)	33.8 (2.7)	36.0 (2.8)	37.7 (2.8)	41.0 (3.8)	<0.001
FFMI, kg/m^2	18.0 [17.0, 18.9]	17.4 [16.4, 18.3]	17.8 [17.0, 18.6]	18.1 [17.1, 19.0]	18.6 [17.6, 19.7]	<0.001	15.2 [14.5, 16.0]	14.0 [13.6, 14.2]	14.8 [14.6, 15.0]	15.5 [15.4, 15.8]	16.6 [16.3, 17.2]	<0.001
FEV_1, L	3.0 (0.7)	3.2 (0.7)	3.0 (0.6)	2.9 (0.6)	2.8 (0.7)	<0.001	2.3 (0.5)	2.2 (0.5)	2.3 (0.5)	2.3 (0.5)	2.4 (0.5)	<0.001
FVC, L	3.8 (0.7)	4.0 (0.7)	3.9 (0.7)	3.7 (0.7)	3.6 (0.7)	<0.001	2.8 (0.5)	2.7 (0.5)	2.8 (0.5)	2.8 (0.5)	2.9 (0.5)	<0.001
Vital capacity, %	101.9 (13.4)	102.7 (13.1)	104.0 (13.3)	101.7 (13.0)	99.3 (13.8)	<0.001	103.1 (13.6)	98.6 (13.4)	102.5 (13.1)	105.2 (13.2)	106.0 (13.6)	0.001
Restrictive ventilatory impairment, %	163 (4.4)	30 (3.2)	23 (2.5)	43 (4.6)	67 (7.2)	<0.001	286 (3.2)	144 (6.5)	70 (3.2)	32 (1.4)	40 (1.8)	<0.001
FEV_1/FVC, %	79.0 (7.1)	79.5 (8.1)	78.5 (6.6)	78.7 (6.8)	79.5 (6.6)	0.902	81.4 (5.8)	81.4 (6.7)	81.2 (6.0)	81.3 (5.4)	81.6 (5.1)	0.094
Obstructive ventilatory impairment, %	314 (8.4)	96 (10.3)	78 (8.4)	82 (8.8)	58 (6.2)	0.016	249 (2.8)	87 (3.9)	67 (3.0)	50 (2.3)	45 (2.0)	<0.001
Education level, %						0.011						0.271

Below high school	2,077 (55.6)	499 (53.4)	502 (53.7)	533 (57.1)	543 (58.1)		4,847 (54.9)	1,192 (54.0)	1,207 (54.8)	1,213 (55.0)	1,235 (56.0)	
Vocational school, junior college, or technical college	456 (12.2)	120 (12.8)	115 (12.3)	106 (11.3)	115 (12.3)		2,850 (32.3)	695 (31.5)	710 (32.2)	719 (32.6)	726 (32.9)	
University or graduate school	1,132 (30.3)	298 (31.9)	308 (33.0)	272 (29.1)	254 (27.2)		1,005 (11.4)	284 (12.9)	259 (11.8)	249 (11.3)	213 (9.7)	
Others	19 (0.5)	9 (1.0)	2 (0.2)	3 (0.3)	5 (0.5)		30 (0.3)	8 (0.4)	8 (0.4)	6 (0.3)	8 (0.4)	
Unknown	52 (1.4)	8 (0.9)	7 (0.7)	20 (2.1)	17 (1.8)		89 (1.0)	27 (1.2)	18 (0.8)	20 (0.9)	24 (1.1)	
Smoking status, %						0.046						0.001
Never smoker	1,076 (28.8)	278 (29.8)	291 (31.2)	254 (27.2)	253 (27.1)		6,924 (78.5)	1,749 (79.3)	1,764 (80.1)	1,758 (79.7)	1,653 (74.9)	
Ex-smoker	1,871 (50.1)	439 (47.0)	456 (48.8)	477 (51.1)	499 (53.4)		1,207 (13.7)	279 (12.6)	283 (12.9)	299 (13.5)	346 (15.7)	
1–19 cigarettes/day	380 (10.2)	114 (12.2)	92 (9.9)	86 (9.2)	88 (9.4)		482 (5.5)	129 (5.8)	105 (4.8)	111 (5.0)	137 (6.2)	
≥20 cigarettes/day	374 (10.0)	99 (10.6)	87 (9.3)	104 (11.1)	84 (9.0)		139 (1.6)	33 (1.5)	29 (1.3)	27 (1.2)	50 (2.3)	
Unknown	35 (0.9)	4 (0.4)	8 (0.9)	13 (1.4)	10 (1.1)		69 (0.8)	16 (0.7)	21 (1.0)	12 (0.5)	20 (0.9)	
Passive smoking, %	864 (23.1)	238 (25.5)	200 (21.4)	202 (21.6)	224 (24.0)	0.294	1,644 (18.6)	372 (16.9)	400 (18.2)	399 (18.1)	473 (21.4)	0.001
Drinking states, %						0.027						0.001
Never drinker	666 (17.8)	155 (16.6)	154 (16.5)	159 (17.0)	198 (21.2)		4,572 (51.8)	1,225 (55.5)	1,136 (51.6)	1,108 (50.2)	1,103 (50.0)	
Ex-drinker	144 (3.9)	40 (4.3)	33 (3.5)	34 (3.6)	37 (4.0)		164 (1.9)	40 (1.8)	42 (1.9)	34 (1.5)	48 (2.2)	
<23 g	1,434 (38.4)	389 (41.6)	369 (39.5)	344 (36.8)	332 (35.5)		3,306 (37.5)	767 (34.8)	862 (39.1)	846 (38.3)	831 (37.7)	
≥23 g	1,468 (39.3)	346 (37.0)	375 (40.1)	386 (41.3)	361 (38.7)		737 (8.4)	165 (7.5)	151 (6.9)	208 (9.4)	213 (9.7)	
Unknown	24 (0.6)	4 (0.4)	3 (0.3)	11 (1.2)	6 (0.6)		42 (0.5)	9 (0.4)	11 (0.5)	11 (0.5)	11 (0.5)	
History of disease, %												
Asthma	226 (6.0)	45 (4.8)	59 (6.3)	54 (5.8)	68 (7.3)	0.156	591 (6.7)	149 (6.8)	128 (5.8)	146 (6.6)	168 (7.6)	0.124

Chronic bronchitis	26 (0.7)	11 (1.2)	4 (0.4)	5 (0.5)	6 (0.6)	0.213	84 (1.0)	18 (0.8)	22 (1.0)	18 (0.8)	26 (1.2)	0.548
COPD	18 (0.5)	5 (0.5)	3 (0.3)	5 (0.5)	5 (0.5)	0.880	9 (0.1)	3 (0.1)	3 (0.1)	1 (0.0)	2 (0.1)	0.747

BF%, body fat percentage; BMI, body mass index; COPD, chronic obstructive pulmonary disease; FEV_1, forced expiratory volume at 1 s; FVC, forced vital capacity; FFM, fat-free mass; FFMI, fat-free mass index; FM, fat mass; FMI, fat mass index; Q, quartile.

Values are expressed as means (standard deviations) or medians (interquartile ranges) for continuous variables, or numbers (%) for categorical variables.

Restrictive ventilatory impairment was defined as reduced VC of <80%.

Obstructive ventilatory impairment was defined as a reduction of FEV_1 indicated by FVC of <70%.

[a] *P* -value for trend test for continuous variables and chi-square test for categorical variables

eTable 2. Participant characteristics by FFMI quartile

FFMI	Men	Q1 (<17.0)	Q2 (17.0–17.9)	Q3 (18.0–18.9)	Q4 (≥18.9)	*P*-value [a]	Women	Q1 (<14.5)	Q2 (14.5–15.1)	Q3 (15.2–16.0)	Q4 (≥16.0)	*P*-value [a]
Number	3,736	927	940	936	933		8,821	2,206	2,202	2,207	2,206	
Age, years	60.1 (14.0)	64.0 (14.1)	61.9 (13.4)	59.8 (13.2)	54.7 (13.6)	<0.001	56.2 (13.4)	56.1 (13.7)	56.4 (13.3)	56.7 (13.3)	55.5 (13.2)	0.295
Height, cm	167.5 (6.3)	166.1 (6.4)	166.8 (6.2)	167.6 (6.1)	169.6 (6.0)	<0.001	155.8 (5.8)	156.0 (5.6)	155.7 (5.9)	155.5 (5.7)	155.9 (5.9)	0.432
BMI, kg/m^2	23.8 (3.1)	21.1 (2.1)	22.9 (1.9)	24.3 (1.9)	26.8 (3.0)	<0.001	22.3 (3.5)	19.5 (1.9)	21.3 (2.1)	22.8 (2.4)	25.8 (3.6)	<0.001
BF%, %	23.7 (6.3)	22.8 (6.7)	23.4 (5.9)	23.7 (5.7)	25.0 (6.6)	<0.001	30.7 (7.3)	28.5 (6.6)	29.6 (6.8)	31.0 (7.0)	33.8 (7.5)	<0.001
FM, kg	16.2 (6.1)	13.6 (5.0)	15.1 (4.9)	16.4 (5.1)	19.7 (7.4)	<0.001	17.1 (6.5)	13.8 (4.3)	15.5 (4.9)	17.4 (5.6)	21.7 (7.6)	<0.001
FMI, kg/m^2	5.5 [4.3, 7.1]	4.8 [3.6, 6.1]	5.3 [4.2, 6.6]	5.7 [4.5, 7.0]	6.6 [5.1, 8.2]	<0.001	6.7 [5.1, 8.6]	5.6 [4.4, 7.0]	6.2 [4.9, 7.8]	7.0 [5.5, 8.7]	8.8 [6.6, 10.8]	<0.001
FFM, kg	50.6 (6.3)	44.6 (4.0)	48.7 (3.7)	51.8 (3.9)	57.4 (5.3)	<0.001	37.1 (4.0)	33.8 (2.7)	36.0 (2.8)	37.7 (2.8)	41.0 (3.8)	<0.001
FFMI, kg/m^2	18.0 [17.0, 18.9]	16.3 [15.8, 16.7]	17.5 [17.2, 17.7]	18.4 [18.2, 18.6]	19.7 [19.2, 20.3]	<0.001	15.2 [14.5, 16.0]	14.0 [13.6, 14.2]	14.8 [14.6, 15.0]	15.5 [15.4, 15.8]	16.6 [16.3, 17.2]	<0.001
FEV_1, L	3.0 (0.7)	2.8 (0.7)	2.9 (0.6)	3.0 (0.6)	3.3 (0.6)	<0.001	2.3 (0.5)	2.2 (0.5)	2.3 (0.5)	2.3 (0.5)	2.4 (0.5)	<0.001
FVC, L	3.8 (0.7)	3.5 (0.7)	3.7 (0.7)	3.8 (0.7)	4.1 (0.7)	<0.001	2.8 (0.5)	2.7 (0.5)	2.8 (0.5)	2.8 (0.5)	2.9 (0.5)	<0.001
Vital capacity, %	101.9 (13.4)	98.2 (13.7)	102.6 (13.0)	103.4 (13.3)	103.5 (12.9)	<0.001	103.1 (13.6)	98.6 (13.4)	102.5 (13.1)	105.2 (13.2)	106.0 (13.6)	<0.001
Restrictive ventilatory impairment, %	163 (4.4)	76 (8.2)	30 (3.2)	29 (3.1)	28 (3.0)	<0.001	286 (3.2)	144 (6.5)	70 (3.2)	32 (1.4)	40 (1.8)	<0.001
FEV_1/FVC, %	79.0 (7.1)	78.2 (8.7)	78.6 (7.2)	78.9 (6.1)	80.4 (5.7)	<0.001	81.4 (5.8)	81.4 (6.7)	81.2 (6.0)	81.3 (5.4)	81.6 (5.1)	0.169
Obstructive ventilatory impairment, %	314 (8.4)	111 (12.0)	94 (10.0)	72 (7.7)	37 (4.0)	<0.001	249 (2.8)	87 (3.9)	67 (3.0)	50 (2.3)	45 (2.0)	<0.001

Education level, %						0.009						0.271
Below high school	2,077 (55.6)	539 (58.1)	524 (55.7)	526 (56.2)	488 (52.3)		4,847 (54.9)	1,192 (54.0)	1,207 (54.8)	1,213 (55.0)	1,235 (56.0)	
Vocational school, junior college, or technical college	456 (12.2)	107 (11.5)	112 (11.9)	96 (10.3)	141 (15.1)		2,850 (32.3)	695 (31.5)	710 (32.2)	719 (32.6)	726 (32.9)	
University or graduate school	1,132 (30.3)	267 (28.8)	276 (29.4)	299 (31.9)	290 (31.1)		1,005 (11.4)	284 (12.9)	259 (11.8)	249 (11.3)	213 (9.7)	
Other	19 (0.5)	6 (0.6)	7 (0.7)	5 (0.5)	1 (0.1)		30 (0.3)	8 (0.4)	8 (0.4)	6 (0.3)	8 (0.4)	
Unknown	52 (1.4)	8 (0.9)	21 (2.2)	10 (1.1)	13 (1.4)		89 (1.0)	27 (1.2)	18 (0.8)	20 (0.9)	24 (1.1)	
Smoking status, %						0.002						0.001
Never smoker	1,076 (28.8)	289 (31.2)	285 (30.3)	263 (28.1)	239 (25.6)		6,924 (78.5)	1,749 (79.3)	1,764 (80.1)	1,758 (79.7)	1,653 (74.9)	
Ex-smoker	1,871 (50.1)	456 (49.2)	464 (49.4)	500 (53.4)	451 (48.3)		1,207 (13.7)	279 (12.6)	283 (12.9)	299 (13.5)	346 (15.7)	
1–19 cigarettes/day	380 (10.2)	97 (10.5)	94 (10.0)	82 (8.8)	107 (11.5)		482 (5.5)	129 (5.8)	105 (4.8)	111 (5.0)	137 (6.2)	
≥20 cigarettes/day	374 (10.0)	76 (8.2)	86 (9.1)	84 (9.0)	128 (13.7)		139 (1.6)	33 (1.5)	29 (1.3)	27 (1.2)	50 (2.3)	
Unknown	35 (0.9)	16 (0.7)	21 (1.0)	12 (0.5)	20 (0.9)		69 (0.8)	16 (0.7)	21 (1.0)	12 (0.5)	20 (0.9)	
Passive smoking, %	864 (23.1)	160 (17.3)	190 (20.2)	229 (24.5)	285 (30.5)	<0.001	1,644 (18.6)	372 (16.9)	400 (18.2)	399 (18.1)	473 (21.4)	0.001
Drinking status, %						0.017						0.001
Never drinker	666 (17.8)	199 (21.5)	145 (15.4)	152 (16.2)	170 (18.2)		4,572 (51.8)	1,225 (55.5)	1,136 (51.6)	1,108 (50.2)	1,103 (50.0)	
Ex-drinker	144 (3.9)	39 (4.2)	41 (4.4)	30 (3.2)	34 (3.6)		164 (1.9)	40 (1.8)	42 (1.9)	34 (1.5)	48 (2.2)	
<23 g	1,434 (38.4)	359 (38.7)	376 (40.0)	358 (38.2)	341 (36.5)		3,306 (37.5)	767 (34.8)	862 (39.1)	846 (38.3)	831 (37.7)	
≥23 g	1,468 (39.3)	324 (35.0)	369 (39.3)	390 (41.7)	385 (41.3)		737 (8.4)	165 (7.5)	151 (6.9)	208 (9.4)	213 (9.7)	
Unknown	24 (0.6)	6 (0.6)	9 (1.0)	6 (0.6)	3 (0.3)		42 (0.5)	9 (0.4)	11 (0.5)	11 (0.5)	11 (0.5)	

History of disease, %												
Asthma	226 (6.0)	54 (5.8)	52 (5.5)	62 (6.6)	58 (6.2)	0.774	591 (6.7)	149 (6.8)	128 (5.8)	146 (6.6)	168 (7.6)	0.124
Chronic bronchitis	26 (0.7)	4 (0.4)	9 (1.0)	8 (0.9)	5 (0.5)	0.465	84 (1.0)	18 (0.8)	22 (1.0)	18 (0.8)	26 (1.2)	0.548
COPD	18 (0.5)	6 (0.6)	2 (0.2)	7 (0.7)	3 (0.3)	0.280	9 (0.1)	3 (0.1)	3 (0.1)	1 (0.0)	2 (0.1)	0.747

BF%, body fat percentage; BMI, body mass index; COPD, chronic obstructive pulmonary disease; FEV_1, forced expiratory volume at 1 s; FVC, forced vital capacity; FFM, fat-free mass; FFMI, fat-free mass index; FM, fat mass; FMI, fat mass index; Q quartile.

Values are expressed as means (standard deviations) or medians (interquartile ranges) for continuous variables, or numbers (%) for categorical variables.

Restrictive ventilatory impairment was defined as reduced VC of <80% of the predicted value.

Obstructive ventilatory impairment was defined as a reduction of FEV_1 indicated by FVC of <70%.

[a] *P*-value for trend test for continuous variables and chi-square test for categorical variables

eTable 3. Adjusted least square means of lung function indices associated by FMI quartile group

Men	Multivariable model						Women	Multivariable model					
FMI	LS means FEV_1	95% CI	*P* for trend	β [a]	SE	*P*	FMI	LS means FEV_1	95% CI	*P* for trend	β [a]	SE	*P*
Q1 (lowest)	3.02	(2.96–3.08)	<0.001	-0.03	0.003	<0.001	Q1 (lowest)	2.30	(2.26–2.34)	<0.001	-0.01	0.001	<0.001
Q2	2.98	(2.91–3.04)					Q2	2.28	(2.25–2.32)				
Q3	2.91	(2.85–2.98)					Q3	2.27	(2.23–2.31)				
Q4 (highest)	2.84	(2.78–2.91)					Q4 (highest)	2.23	(2.20–2.27)				
Men	Multivariable model						Women	Multivariable model					
FMI	LS means FVC	95% CI	*P* for trend	β [a]	SE	*P*	FMI	LS means FVC	95% CI	*P* for trend	β [a]	SE	*P*
Q1 (lowest)	3.85	(3.78–3.93)	<0.001	-0.05	0.004	<0.001	Q1 (lowest)	2.86	(2.81–2.90)	<0.001	-0.02	0.002	<0.001
Q2	3.82	(3.75–3.90)					Q2	2.84	(2.80–2.89)				
Q3	3.71	(3.64–3.79)					Q3	2.82	(2.78–2.87)				
Q4 (highest)	3.59	(3.51–3.67)					Q4 (highest)	2.74	(2.70–2.79)				

CI, confidence interval; FEV_1, forced expiratory volume at 1 s; FMI, fat mass index; FVC, forced vital capacity; LS, least squares; Q, quartile.

Adjusted for age, education level (below high school; vocational school, junior college, or technical college; university or graduate school; other; unknown), smoking status (never smoker, ex-smoker, current smoker [1–19 or ≥20 cigarettes/day]), unknown), passive smoking (yes, no), and drinking status (never drinker, ex-drinker, current drinker [<23 or ≥23 g/day]), unknown).

[a] Multiple regression analysis with actual FMI as continuous variables.

eTable 4. Adjusted least square means of lung function indices according to FFMI quartile

Men	Multivariable model						Women	Multivariable model					
FFMI	LS means FEV_1	95% CI	*P* for trend	β [a]	SE	*P*	FFMI	LS means FEV_1	95% CI	*P* for trend	β [a]	SE	*P*
Q1 (lowest)	2.84	(2.78–2.90)	<0.001	0.05	0.01	<0.001	Q1 (lowest)	2.19	(2.15–2.22)	<0.001	0.04	0.003	<0.001
Q2	2.94	(2.88–3.00)					Q2	2.26	(2.22–2.29)				
Q3	2.97	(2.90–3.03)					Q3	2.30	(2.27–2.34)				
Q4 (highest)	3.04	(2.98–3.11)					Q4 (highest)	2.33	(2.29–2.36)				
Men	Multivariable model						Women	Multivariable model					
FFMI	LS means FVC	95% CI	*P* for trend	β [a]	SE	*P*	FFMI	LS means FVC	95% CI	*P* for trend	β [a]	SE	*P*
Q1 (lowest)	3.62	(3.54–3.69)	<0.001	0.06	0.01	<0.001	Q1 (lowest)	2.71	(2.66–2.75)	<0.001	0.05	0.004	<0.001
Q2	3.75	(3.68–3.83)					Q2	2.80	(2.76–2.85)				
Q3	3.79	(3.71–3.87)					Q3	2.86	(2.81–2.90)				
Q4 (highest)	3.85	(3.77–3.93)					Q4 (highest)	2.88	(2.83–2.92)				

CI, confidence interval; FEV_1, forced expiratory volume at 1 s; FFMI, fat-free mass index; FVC, forced vital capacity; LS, least squares; Q, quartile.

Adjusted for age, education level (below high school; vocational school, junior college, or technical college; university or graduate school; other; unknown), smoking status (never smoker, ex-smoker, current smoker [1–19 or ≥20 cigarettes/day]), unknown), passive smoking (yes, no), and drinking status (never drinker, ex-drinker, current drinker [<23 or ≥23 g/day]), unknown).

[a] Multiple regression analysis with actual FFMI as continuous variables.

eTable 5. Association of combined FMI and FFMI with FEV_1 among never smokers

Men	FMI Q1			FMI Q2			FMI Q3			FMI Q4			Multiple linear regression of FFMI subgroups [b]		
	LS means FEV_1 (L), 95% CI												β	SE	*P*
FFMI Q1	3.06	(2.92–3.20)		2.91	(2.76–3.06)		2.78	(2.63–2.94)		2.70	(2.52–2.87)		-0.08	0.01	<0.001
FFMI Q2	3.15	(3.00–3.29)		3.08	(2.94–3.22)		2.95	(2.80–3.10)		2.86	(2.70–3.03)		-0.06	0.02	<0.001
FFMI Q3	3.18	(3.02–3.33)		3.05	(2.90–3.20)		3.04	(2.89–3.18)		2.90	(2.75–3.05)		-0.05	0.01	<0.001
FFMI Q4	3.31	(3.13–3.49)		3.24	(3.08–3.40)		3.10	(2.95–3.25)		3.03	(2.90–3.16)		-0.04	0.01	0.002
Multiple linear regression of FMI subgroups [a]	β	SE	*P*	β	SE	*P*	β	SE	*P*	β	SE	*P*			
	0.06	0.02	0.003	0.09	0.09	<0.001	0.07	0.02	<0.001	0.08	0.02	<0.001			
Women	LS means FEV_1 (L), 95% CI												Multiple linear regression of FFMI subgroups [b]		
FFMI Q1	2.12	(2.04–2.21)		2.09	(2.01–2.18)		2.07	(1.98–2.15)		2.04	(1.94–2.14)		-0.02	0.004	<0.001
FFMI Q2	2.21	(2.12–2.29)		2.19	(2.11–2.28)		2.15	(2.06–2.24)		2.07	(1.98–2.16)		-0.02	0.004	<0.001
FFMI Q3	2.28	(2.19–2.37)		2.25	(2.16–2.34)		2.22	(2.13–2.30)		2.11	(2.03–2.20)		-0.03	0.003	<0.001
FFMI Q4	2.35	(2.25–2.44)		2.30	(2.21–2.39)		2.26	(2.18–2.35)		2.18	(2.10–2.26)		-0.02	0.003	<0.001
Multiple linear regression of FMI subgroups [a]	β	SE	*P*	β	SE	*P*	β	SE	*P*	β	SE	*P*			
	0.09	0.01	<0.001	0.08	0.01	<0.001	0.08	0.01	<0.001	0.04	0.01	<0.001			

CI, confidence interval; FEV_1, forced expiratory volume at 1 s; FFMI, fat-free mass index; FMI, fat mass index; LS, least squares; Q, quartile.

Adjusted for age, education level (below high school; vocational school, junior college, or technical college; university or graduate school; other; unknown), passive smoking (yes, no), and drinking status (never drinker, ex-drinker, current drinker [<23 or ≥23 g/day]), unknown).

[a] Multiple regression analysis with stratified FMI and actual FFMI as continuous variables.

[b] Multiple regression analysis with stratified FFMI and actual FMI as continuous variables.

eTable 6. Association of combined FMI and FFMI with FVC among never smokers

Men	FMI Q1			FMI Q2			FMI Q3			FMI Q4			Multiple linear regression of FFMI subgroups [b]		
	LS means FVC (L), 95% CI												β	SE	*P*
FFMI Q1	3.78	(3.61–3.94)		3.60	(3.43–3.78)		3.44	(3.25–3.63)		3.34	(3.13–3.55)		-0.10	0.02	<0.001
FFMI Q2	3.92	(3.75–4.09)		3.87	(3.70–4.05)		3.73	(3.55–3.90)		3.49	(3.30–3.69)		-0.08	0.02	<0.001
FFMI Q3	3.95	(3.77–4.14)		3.85	(3.67–4.02)		3.80	(3.63–3.98)		3.56	(3.38–3.75)		-0.07	0.02	<0.001
FFMI Q4	4.19	(3.98–4.40)		4.03	(3.84–4.22)		3.88	(3.71–4.06)		3.75	(3.59–3.91)		-0.06	0.01	<0.001
Multiple linear regression of FMI subgroups [a]	β	SE	*P*	β	SE	*P*	β	SE	*P*	β	SE	*P*			
	0.08	0.02	<0.001	0.13	0.03	<0.001	0.09	0.02	<0.001	0.10	0.03	<0.001			
Women	LS means FVC (L), 95% CI												Multiple linear regression of FFMI subgroups [b]		
FFMI Q1	2.58	(2.48–2.68)		2.57	(2.47–2.68)		2.54	(2.44–2.64)		2.47	(2.35–2.59)		-0.02	0.01	<0.001
FFMI Q2	2.72	(2.61–2.82)		2.68	(2.58–2.78)		2.64	(2.54–2.74)		2.50	(2.40–2.61)		-0.03	0.004	<0.001
FFMI Q3	2.81	(2.70–2.91)		2.78	(2.68–2.89)		2.71	(2.61–2.81)		2.55	(2.45–2.65)		-0.02	0.004	<0.001
FFMI Q4	2.90	(2.79–3.01)		2.85	(2.74–2.95)		2.77	(2.67–2.88)		2.64	(2.54–2.74)		-0.04	0.003	0.002
Multiple linear regression of FMI subgroups [a]	β	SE	*P*	β	SE	*P*	β	SE	*P*	β	SE	*P*			
	0.12	0.01	<0.001	0.11	0.01	<0.001	0.09	0.01	<0.001	0.04	0.01	<0.001			

CI, confidence interval; FFMI, fat-free mass index; FMI, fat mass index; FVC, forced vital capacity; LS, least squares; Q, quartile.

Adjusted for age, education level (below high school; vocational school, junior college, or technical college; university or graduate school; other; unknown), passive smoking (yes, no), and drinking status (never drinker, ex-drinker, current drinker [<23 or ≥23 g/day]), unknown).

[a] Multiple regression analysis with stratified FMI and actual FFMI as continuous variables.

[b] Multiple regression analysis with stratified FFMI and actual FMI as continuous variables.

eTable 7. Association of combined FMI and FFMI with FEV_1 among the lowest age tertile

Men[a]	FMI Q1			FMI Q2			FMI Q3			FMI Q4			Multiple linear regression of FFMI subgroups [c]		
	LS means FEV_1 (L), 95% CI												β	SE	*P*
FFMI Q1	3.61	(3.47–3.75)		3.46	(3.31–3.61)		3.49	(3.33–3.65)		3.42	(3.17–3.67)		-0.04	0.02	0.022
FFMI Q2	3.73	(3.58–3.89)		3.68	(3.53–3.83)		3.59	(3.44–3.75)		3.41	(3.24–3.58)		-0.06	0.01	<0.001
FFMI Q3	3.87	(3.70–4.03)		3.72	(3.57–3.88)		3.62	(3.46–3.77)		3.62	(3.46–3.78)		-0.06	0.01	0.003
FFMI Q4	3.97	(3.76–4.19)		3.84	(3.66–4.02)		3.75	(3.59–3.91)		3.60	(3.46–3.74)		-0.05	0.01	<0.001
Multiple linear regression of FMI subgroups [b]	β	SE	*P*	β	SE	*P*	β	SE	*P*	β	SE	*P*			
	0.11	0.02	<0.001	0.10	0.02	<0.001	0.07	0.02	<0.001	0.03	0.02	<0.001			
Women [a]	LS means FEV_1 (L), 95% CI												Multiple linear regression of FFMI subgroups [c]		
FFMI Q1	2.59	(2.51–2.67)		2.57	(2.48–2.65)		2.49	(2.40–2.58)		2.44	(2.31–2.56)		-0.03	0.01	<0.001
FFMI Q2	2.68	(2.60–2.76)		2.64	(2.56–2.72)		2.67	(2.58–2.75)		2.60	(2.49–2.70)		-0.01	0.01	0.115
FFMI Q3	2.72	(2.63–2.81)		2.72	(2.63–2.80)		2.67	(2.58–2.75)		2.63	(2.54–2.71)		-0.02	0.01	0.002
FFMI Q4	2.83	(2.72–2.94)		2.83	(2.73–2.93)		2.77	(2.68–2.86)		2.66	(2.58–2.74)		-0.02	0.004	<0.001
Multiple linear regression of FMI subgroups [b]	β	SE	*P*	β	SE	*P*	β	SE	*P*	β	SE	*P*			
	0.08	0.01	<0.001	0.10	0.01	<0.001	0.10	0.01	<0.001	0.04	0.01	<0.001			

CI, confidence interval; FEV_1, forced expiratory volume at 1 s; FFMI, fat-free mass index; FMI, fat mass index; LS, least squares; Q, quartile.

Adjusted for age, education level (below high school; vocational school, junior college, or technical college; university or graduate school; other; unknown), smoking status (never smoker, ex-smoker, current smoker [1–19 or ≥20 cigarettes/day]), unknown), passive smoking (yes, no), and drinking status (never drinker, ex-drinker, current drinker [<23 or ≥23 g/day]), unknown).

[a] Men <58 years old and women <52 years old

[b] Multiple regression analysis with stratified FMI and actual FFMI as continuous variables.

[c] Multiple regression analysis with stratified FFMI and actual FMI as continuous variables.

eTable 8. Association of combined FMI and FFMI with FEV_1 among the second age tertile

Men[a]	FMI Q1			FMI Q2			FMI Q3			FMI Q4			Multiple linear regression of FFMI subgroups [c]		
	LS means FEV_1 (L), 95% CI												β	SE	*P*
FFMI Q1	2.81	(2.65–2.96)		2.76	(2.59–2.93)		2.65	(2.47–2.84)		2.50	(2.30–2.70)		-0.05	0.02	0.011
FFMI Q2	2.94	(2.77–3.11)		2.87	(2.71–3.04)		2.70	(2.52–2.87)		2.68	(2.51–2.86)		-0.07	0.01	<0.001
FFMI Q3	2.86	(2.67–3.05)		2.83	(2.65–3.00)		2.84	(2.68–3.00)		2.83	(2.66–2.99)		0.00	0.01	0.873
FFMI Q4	2.99	(2.79–3.20)		2.96	(2.77–3.14)		2.91	(2.75–3.08)		2.77	(2.62–2.93)		-0.04	0.01	<0.001
Multiple linear regression of FMI subgroups [b]	β	SE	*P*	β	SE	*P*	β	SE	*P*	β	SE	*P*			
	0.05	0.02	0.022	0.07	0.02	0.002	0.09	0.02	<0.001	0.06	0.02	0.003			
Women [a]	LS means FEV_1 (L), 95% CI												Multiple linear regression of FFMI subgroups [c]		
FFMI Q1	2.22	(2.14–2.29)		2.13	(2.05–2.20)		2.17	(2.09–2.25)		2.13	(2.01–2.25)		-0.02	0.01	0.007
FFMI Q2	2.29	(2.21–2.37)		2.28	(2.20–2.35)		2.23	(2.15–2.30)		2.15	(2.06–2.24)		-0.03	0.01	<0.001
FFMI Q3	2.38	(2.30–2.46)		2.32	(2.24–2.40)		2.31	(2.23–2.39)		2.20	(2.13–2.28)		-0.03	0.01	<0.001
FFMI Q4	2.49	(2.39–2.58)		2.39	(2.30–2.47)		2.30	(2.22–2.38)		2.25	(2.18–2.33)		-0.02	0.004	<0.001
Multiple linear regression of FMI subgroups [b]	β	SE	*P*	β	SE	*P*	β	SE	*P*	β	SE	*P*			
	0.10	0.01	<0.001	0.09	0.01	<0.001	0.06	0.01	<0.001	0.03	0.01	<0.001			

CI, confidence interval; FEV_1, forced expiratory volume at 1 s; FFMI, fat-free mass index; FMI, fat mass index; LS, least squares; Q, quartile.

Adjusted for age, education level (below high school; vocational school, junior college, or technical college; university or graduate school; other; unknown), smoking status (never smoker, ex-smoker, current smoker [1–19 or ≥20 cigarettes/day]), unknown), passive smoking (yes, no), and drinking status (never drinker, ex-drinker, current drinker [<23 or ≥23 g/day]), unknown).

[a] Men aged 58–67 years and women aged 52–64 years

[b] Multiple regression analysis with stratified FMI and actual FFMI as continuous variables.

[c] Multiple regression analysis with stratified FFMI and actual FMI as continuous variables.

eTable 9. Association of combined FMI and FFMI with FEV_1 among the highest age tertile

Men[a]	FMI Q1			FMI Q2			FMI Q3			FMI Q4			Multiple linear regression of FFMI subgroups [c]		
	LS means FEV_1 (L), 95% CI												β	SE	*P*
FFMI Q1	2.43	(2.32–2.54)		2.32	(2.20–2.44)		2.27	(2.14–2.40)		2.17	(2.02–2.32)		-0.06	0.01	<0.001
FFMI Q2	2.59	(2.46–2.71)		2.50	(2.38–2.63)		2.36	(2.23–2.48)		2.26	(2.13–2.39)		-0.07	0.01	<0.001
FFMI Q3	2.59	(2.47–2.72)		2.52	(2.40–2.64)		2.36	(2.24–2.49)		2.30	(2.18–2.42)		-0.07	0.01	<0.001
FFMI Q4	2.71	(2.56–2.85)		2.58	(2.44–2.72)		2.51	(2.39–2.63)		2.42	(2.31–2.53)		-0.04	0.01	<0.001
Multiple linear regression of FMI subgroups [b]	β	SE	*P*	β	SE	*P*	β	SE	*P*	β	SE	*P*			
	0.06	0.02	<0.001	0.08	0.02	<0.001	0.07	0.02	<0.001	0.07	0.02	<0.001			
Women [a]	LS means FEV_1 (L), 95% CI												Multiple linear regression of FFMI subgroups [c]		
FFMI Q1	1.88	(1.82–1.95)		1.89	(1.82–1.96)		1.83	(1.76–1.91)		1.79	(1.68–1.90)		-0.01	0.01	0.019
FFMI Q2	1.97	(1.90–2.04)		1.92	(1.85–1.99)		1.87	(1.80–1.94)		1.84	(1.76–1.92)		-0.02	0.01	<0.001
FFMI Q3	2.00	(1.92–2.08)		2.01	(1.94–2.08)		1.94	(1.87–2.02)		1.87	(1.80–1.94)		-0.03	0.004	<0.001
FFMI Q4	1.96	(1.88–2.04)		2.05	(1.98–2.13)		2.04	(1.96–2.11)		1.92	(1.85–1.98)		-0.02	0.004	<0.001
Multiple linear regression of FMI subgroups [b]	β	SE	*P*	β	SE	*P*	β	SE	*P*	β	SE	*P*			
	0.05	0.01	<0.001	0.07	0.01	<0.001	0.07	0.02	<0.001	0.04	0.01	<0.001			

CI, confidence interval; FEV1, forced expiratory volume at 1 s; FFMI, fat-free mass index; FMI, fat mass index; LS, least squares; Q, quartile.

Adjusted for age, education level (below high school; vocational school, junior college, or technical college; university or graduate school; other; unknown), smoking status (never smoker, ex-smoker, current smoker [1–19 or ≥20 cigarettes/day]), unknown), passive smoking (yes, no), and drinking status (never drinker, ex-drinker, current drinker [<23 or ≥23 g/day]), unknown).

[a] Men >67 years old and women >64 years old

[b] Multiple regression analysis with stratified FMI and actual FFMI as continuous variables.

[c] Multiple regression analysis with stratified FFMI and actual FMI as continuous variables.

eTable 10. Association of combined FMI and FFMI with FVC among the lowest age tertile

Men[a]	FMI Q1			FMI Q2			FMI Q3			FMI Q4			Multiple linear regression of FFMI subgroups [c]		
	LS means FVC (L), 95% CI												β	SE	*P*
FFMI Q1	4.32	(4.15–4.49)		4.22	(4.04–4.40)		4.27	(4.08–4.46)		4.15	(3.86–4.45)		-0.03	0.02	0.210
FFMI Q2	4.63	(4.45–4.82)		4.51	(4.33–4.69)		4.41	(4.23–4.60)		4.15	(3.94–4.35)		-0.09	0.02	<0.001
FFMI Q3	4.77	(4.57–4.97)		4.56	(4.37–4.74)		4.43	(4.24–4.61)		4.43	(4.23–4.62)		-0.06	0.02	<0.001
FFMI Q4	4.97	(4.72–5.23)		4.72	(4.50–4.94)		4.58	(4.39–4.77)		4.37	(4.21–4.54)		-0.06	0.01	<0.001
Multiple linear regression of FMI subgroups [b]	β	SE	*P*	β	SE	*P*	β	SE	*P*	β	SE	*P*			
	0.18	0.02	<0.001	0.14	0.02	<0.001	0.08	0.02	<0.001	0.04	0.02	<0.001			
Women [a]	LS means FVC (L), 95% CI												Multiple linear regression of FFMI subgroups [c]		
FFMI Q1	3.05	(2.96–3.14)		3.06	(2.96–3.16)		3.00	(2.90–3.10)		2.95	(2.81–3.10)		-0.03	0.01	<0.001
FFMI Q2	3.21	(3.11–3.30)		3.18	(3.08–3.27)		3.19	(3.09–3.29)		3.12	(3.01–3.24)		-0.01	0.01	0.115
FFMI Q3	3.30	(3.20–3.40)		3.27	(3.17–3.37)		3.23	(3.14–3.33)		3.15	(3.05–3.25)		-0.02	0.01	<0.001
FFMI Q4	3.47	(3.34–3.60)		3.43	(3.32–3.54)		3.35	(3.25–3.45)		3.21	(3.12–3.30)		-0.02	0.004	<0.001
Multiple linear regression of FMI subgroups [b]	β	SE	*P*	β	SE	*P*	β	SE	*P*	β	SE	*P*			
	0.14	0.02	<0.001	0.13	0.02	<0.001	0.13	0.01	<0.001	0.04	0.01	0.002			

CI, confidence interval; FFMI, fat-free mass index; FMI, fat mass index; FVC, forced vital capacity; LS, least squares; Q, quartile.

Adjusted for age, education level (below high school; vocational school, junior college, or technical college; university or graduate school; other; unknown), smoking status (never smoker, ex-smoker, current smoker [1–19 or ≥20 cigarettes/day]), unknown), passive smoking (yes, no), and drinking status (never drinker, ex-drinker, current drinker [<23 or ≥23 g/day]), unknown).

[a] Men <58 years old and women <52 years old

[b] Multiple regression analysis with stratified FMI and actual FFMI as continuous variables.

[c] Multiple regression analysis with stratified FFMI and actual FMI as continuous variables.

eTable 11. Association of combined FMI and FFMI with FVC among the second age tertile

Men[a]	FMI Q1			FMI Q2			FMI Q3			FMI Q4			Multiple linear regression of FFMI subgroups [c]		
	LS means FVC (L), 95% CI												β	SE	*P*
FFMI Q1	3.68	(3.49–3.86)		3.66	(3.45–3.86)		3.49	(3.27–3.71)		3.31	(3.07–3.55)		-0.06	0.02	0.009
FFMI Q2	3.81	(3.61–4.01)		3.76	(3.56–3.95)		3.54	(3.33–3.75)		3.48	(3.27–3.69)		-0.08	0.02	<0.001
FFMI Q3	3.80	(3.57–4.02)		3.74	(3.53–3.94)		3.71	(3.52–3.91)		3.61	(3.41–3.81)		-0.03	0.02	0.057
FFMI Q4	3.92	(3.68–4.16)		3.88	(3.66–4.10)		3.77	(3.57–3.97)		3.58	(3.39–3.77)		-0.06	0.01	<0.001
Multiple linear regression of FMI subgroups [b]	β	SE	*P*	β	SE	*P*	β	SE	*P*	β	SE	*P*			
	0.08	0.02	<0.001	0.08	0.03	0.005	0.09	0.03	<0.001	0.06	0.02	0.008			
Women [a]	LS means FVC (L), 95% CI												Multiple linear regression of FFMI subgroups [c]		
FFMI Q1	2.76	(2.68–2.85)		2.68	(2.59–2.77)		2.71	(2.62–2.81)		2.66	(2.52–2.81)		-0.02	0.01	0.030
FFMI Q2	2.87	(2.78–2.97)		2.86	(2.77–2.95)		2.80	(2.70–2.89)		2.65	(2.55–2.76)		-0.03	0.01	<0.001
FFMI Q3	3.01	(2.91–3.11)		2.93	(2.83–3.03)		2.88	(2.78–2.97)		2.73	(2.63–2.82)		-0.04	0.01	<0.001
FFMI Q4	3.12	(3.01–3.23)		2.98	(2.87–3.08)		2.87	(2.77–2.96)		2.76	(2.68–2.85)		-0.04	0.004	<0.001
Multiple linear regression of FMI subgroups [b]	β	SE	*P*	β	SE	*P*	β	SE	*P*	β	SE	*P*			
	0.14	0.01	<0.001	0.11	0.01	<0.001	0.07	0.01	<0.001	0.03	0.01	0.023			

CI, confidence interval; FFMI, fat-free mass index; FMI, fat mass index; FVC, forced vital capacity; LS, least squares; Q, quartile.
Adjusted for age, education level (below high school; vocational school, junior college, or technical college; university or graduate school; other; unknown), smoking status (never smoker, ex-smoker, current smoker [1–19 or ≥20 cigarettes/day]), unknown), passive smoking (yes, no), and drinking status (never drinker, ex-drinker, current drinker [<23 or ≥23 g/day]), unknown).

[a] Men aged 58–67 years and women aged 52–64 years

[b] Multiple regression analysis with stratified FMI and actual FFMI as continuous variables.

[c] Multiple regression analysis with stratified FFMI and actual FMI as continuous variables.

eTable 12. Association of combined FMI and FFMI with FVC among the highest age tertile

Men[a]	FMI Q1			FMI Q2			FMI Q3			FMI Q4			Multiple linear regression of FFMI subgroups [c]		
	LS means FVC (L), 95% CI												β	SE	*P*
FFMI Q1	3.28		(3.15–3.41)	3.12		(2.98–3.26)	3.08		(2.93–3.23)	2.91		(2.74–3.08)	-0.07	0.02	<0.001
FFMI Q2	3.47		(3.33–3.61)	3.37		(3.23–3.51)	3.13		(2.99–3.27)	2.98		(2.84–3.13)	-0.11	0.02	<0.001
FFMI Q3	3.48		(3.33–3.62)	3.37		(3.23–3.51)	3.15		(3.00–3.29)	3.03		(2.89–3.17)	-0.10	0.01	<0.001
FFMI Q4	3.62		(3.46–3.79)	3.42		(3.26–3.57)	3.31		(3.17–3.44)	3.16		(3.03–3.29)	-0.07	0.01	<0.001
Multiple linear regression of	β	SE	*P*	β	SE	*P*	β	SE	*P*	β	SE	*P*			
FMI subgroups b	0.09	0.02	<0.001	0.10	0.02	<0.001	0.07	0.02	<0.001	0.08	0.02	<0.001			
Women [a]	LS means FVC (L), 95% CI												Multiple linear regression of FFMI subgroups [c]		
FFMI Q1	2.43		(2.35–2.51)	2.44		(2.35–2.52)	2.36		(2.27–2.45)	2.26		(2.13–2.39)	-0.02	0.01	0.001
FFMI Q2	2.55		(2.46–2.63)	2.47		(2.39–2.56)	2.40		(2.32–2.49)	2.34		(2.25–2.43)	-0.03	0.01	<0.001
FFMI Q3	2.60		(2.50–2.69)	2.56		(2.48–2.65)	2.47		(2.39–2.56)	2.36		(2.28–2.45)	-0.03	0.01	<0.001
FFMI Q4	2.54		(2.44–2.64)	2.66		(2.57–2.75)	2.57		(2.48–2.65)	2.42		(2.34–2.49)	-0.03	0.004	0.003
Multiple linear regression of	β	SE	*P*	β	SE	*P*	β	SE	*P*	β	SE	*P*			
FMI subgroups b	0.07	0.01	<0.001	0.08	0.01	<0.001	0.08	0.01	<0.001	0.04	0.01	0.001			

CI, confidence interval; FFMI, fat-free mass index; FMI, fat mass index; FVC, forced vital capacity; LS, least squares; Q, quartile.

Adjusted for age, education level (below high school; vocational school, junior college, or technical college; university or graduate school; other; unknown), smoking status (never smoker, ex-smoker, current smoker [1–19 or ≥20 cigarettes/day]), unknown), passive smoking (yes, no), and drinking status (never drinker, ex-drinker, current drinker [<23 or ≥23 g/day]), unknown).

[a] Men >67 years old and women >64 years old

[b] Multiple regression analysis with stratified FMI and actual FFMI as continuous variables.

[c] Multiple regression analysis with stratified FFMI and actual FMI as continuous variables.

eTable 13. Association of combined FMI and FFMI with FEV_1 among participants without respiratory disease

Men	FMI Q1			FMI Q2			FMI Q3			FMI Q4			Multiple linear regression of FFMI subgroups [b]		
	LS means FEV_1 (L), 95% CI												β	SE	*P*
FFMI Q1	3.12	(3.05–3.19)		3.03	(2.95–3.10)		2.97	(2.89–3.06)		2.86	(2.76–2.96)		-0.06	0.01	<0.001
FFMI Q2	3.15	(3.08–3.23)		3.10	(3.03–3.18)		3.00	(2.92–3.08)		2.94	(2.86–3.03)		-0.05	0.08	<0.001
FFMI Q3	3.19	(3.10–3.27)		3.12	(3.04–3.20)		3.12	(3.05–3.20)		2.99	(2.91–3.07)		-0.04	0.01	<0.001
FFMI Q4	3.35	(3.25–3.44)		3.26	(3.18–3.35)		3.16	(3.08–3.24)		3.07	(3.00–3.14)		-0.04	0.01	<0.001
Multiple linear regression of FMI subgroups [a]	β	SE	*P*	β	SE	*P*	β	SE	*P*	β	SE	*P*			
	0.05	0.01	<0.001	0.07	0.01	<0.001	0.06	0.01	<0.001	0.04	0.01	<0.001			
**Women**	LS means FEV_1 (L), 95% CI												Multiple linear regression of FFMI subgroups [b]		
FFMI Q1	2.29	(2.25–2.33)		2.25	(2.21–2.30)		2.21	(2.17–2.26)		2.21	(2.15–2.27)		-0.02	0.004	<0.001
FFMI Q2	2.35	(2.31–2.39)		2.33	(2.29–2.38)		2.28	(2.24–2.33)		2.21	(2.16–2.26)		-0.02	0.003	<0.001
FFMI Q3	2.40	(2.36–2.45)		2.36	(2.32–2.41)		2.35	(2.31–2.40)		2.26	(2.22–2.30)		-0.02	0.003	<0.001
FFMI Q4	2.50	(2.44–2.55)		2.43	(2.38–2.47)		2.39	(2.35–2.44)		2.31	(2.27–2.34)		-0.02	0.002	<0.001
Multiple linear regression of FMI subgroups [a]	β	SE	*P*	β	SE	*P*	β	SE	*P*	β	SE	*P*			
	0.07	0.01	<0.001	0.06	0.01	<0.001	0.07	0.01	<0.001	0.03	0.01	<0.001			

CI, confidence interval; FEV_1, forced expiratory volume at 1 s; FFMI, fat-free mass index; FMI, fat mass index; LS, least squares; Q, quartile.

Adjusted for age, education level (below high school; vocational school, junior college, or technical college; university or graduate school; other; unknown), smoking status (never smoker, ex-smoker, current smoker [1–19 or ≥20 cigarettes/day]), unknown), passive smoking (yes, no), and drinking status (never drinker, ex-drinker, current drinker [<23 or ≥23 g/day]), unknown).

[a] Multiple regression analysis with stratified FMI and actual FFMI as continuous variables.

[b] Multiple regression analysis with stratified FFMI and actual FMI as continuous variables.

eTable 14. Association of combined FMI and FFMI with FVC among participants without respiratory disease

Men	FMI Q1			FMI Q2			FMI Q3			FMI Q4			Multiple linear regression of FFMI subgroups [b]		
	LS means FVC (L), 95% CI												β	SE	*P*
FFMI Q1	3.83	(3.74–3.92)		3.78	(3.69–3.88)		3.68	(3.58–3.79)		3.52	(3.39–3.64)		-0.06	0.01	<0.001
FFMI Q2	3.90	(3.81–4.00)		3.88	(3.78–3.98)		3.74	(3.64–3.84)		3.64	(3.54–3.75)		-0.06	0.01	<0.001
FFMI Q3	4.00	(3.90–4.11)		3.93	(3.83–4.03)		3.89	(3.80–3.99)		3.70	(3.60–3.80)		-0.06	0.01	<0.001
FFMI Q4	4.21	(4.09–4.32)		4.08	(3.97–4.19)		3.91	(3.81–4.01)		3.79	(3.70–3.88)		-0.06	0.01	<0.001
Multiple linear regression of FMI subgroups [a]	β	SE	*P*	β	SE	*P*	β	SE	*P*	β	SE	*P*			
	0.09	0.01	<0.001	0.09	0.01	<0.001	0.07	0.01	<0.001	0.05	0.01	<0.001			
Women	LS means FVC (L), 95% CI												Multiple linear regression of FFMI subgroups [b]		
FFMI Q1	2.80	(2.76–2.85)		2.79	(2.74–2.84)		2.74	(2.69–2.80)		2.70	(2.63–2.78)		-0.02	0.004	<0.001
FFMI Q2	2.91	(2.86–2.96)		2.88	(2.83–2.93)		2.84	(2.78–2.89)		2.70	(2.64–2.76)		-0.03	0.004	<0.001
FFMI Q3	2.98	(2.92–3.03)		2.94	(2.89–2.99)		2.91	(2.85–2.96)		2.76	(2.71–2.81)		-0.03	0.004	<0.001
FFMI Q4	3.12	(3.06–3.18)		3.02	(2.96–3.08)		2.96	(2.90–3.01)		2.82	(2.77–2.87)		-0.04	0.003	<0.001
Multiple linear regression of FMI subgroups [a]	β	SE	*P*	β	SE	*P*	β	SE	*P*	β	SE	*P*			
	0.11	0.01	<0.001	0.09	0.01	<0.001	0.08	0.01	<0.001	0.03	0.01	<0.001			

CI, confidence interval; FFMI, fat-free mass index; FMI, fat mass index; FVC, forced vital capacity; LS, least squares; Q, quartile.

Adjusted for age, education level (below high school; vocational school, junior college, or technical college; university or graduate school; other; unknown), smoking status (never smoker, ex-smoker, current smoker [1–19 or ≥20 cigarettes/day]), unknown), passive smoking (yes, no), and drinking status (never drinker, ex-drinker, current drinker [<23 or ≥23 g/day]), unknown.

[a] Multiple regression analysis with stratified FMI and actual FFMI as continuous variables.

[b] Multiple regression analysis with stratified FFMI and actual FMI as continuous variables.
